# Production of the antidepressant orcinol glucoside in *Yarrowia lipolytica* with yields over 6,400-fold higher than plant extraction

**DOI:** 10.1371/journal.pbio.3002131

**Published:** 2023-06-06

**Authors:** Bihuan Chen, Xiaonan Liu, Yina Wang, Jie Bai, Xiangyu Liu, Guisheng Xiang, Wei Liu, Xiaoxi Zhu, Jian Cheng, Lina Lu, Guanghui Zhang, Ge Zhang, Zongjie Dai, Shuhui Zi, Shengchao Yang, Huifeng Jiang

**Affiliations:** 1 College of Agronomy and Biotechnology, National and Local Joint Engineering Research Center on Germplasm Innovation & Utilization of Chinese Medicinal Materials in Southwest China, Key Laboratory of Medicinal Plant Biology of Yunnan Province, Yunnan Agricultural University, Kunming, Yunnan, China; 2 Key Laboratory of Engineering Biology for Low-Carbon Manufacturing, Tianjin Institute of Industrial Biotechnology, Chinese Academy of Sciences, Tianjin, China; 3 National Center of Technology Innovation for Synthetic Biology, Tianjin, China; 4 Yunnan Characteristic Plant Extraction Laboratory, Kunming, Yunnan, China; 5 University of Chinese Academy of Sciences, Beijing, China; 6 College of Biotechnology, Tianjin University of Science and Technology, Tianjin, China; 7 College of Life Science and Technology, Wuhan Polytechnic University, Wuhan, Hubei, China; Zhejiang University, CHINA

## Abstract

Orcinol glucoside (OG), mainly found in the rhizome of the traditional Chinese herb *Curculigo orchioides* Gaertn, is noted for its antidepressant effects. In this study, an efficient screening pipeline was established for identifying the highly active orcinol synthase (ORS) and UDP-dependent glycosyltransferase (UGT) involved in the biosynthesis of OG by combining transcriptome analysis, structure-based virtual screening, and in vitro enzyme activity assays. By enhancing the downstream pathway, metabolic engineering and fermentation optimization, the OG production in *Yarrowia lipolytica* was improved 100-fold, resulting in a final yield of 43.46 g/L (0.84 g/g DCW), which is almost 6,400-fold higher than the extraction yield from *C*. *orchioides* roots. This study provides a reference for rapid identification of functional genes and high-yield production of natural products.

## Abstract 2

This study deciphers the biosynthetic pathway of the antidepressant orcinol glucoside from Curculigo orchioides and engineers the production of orcinol glucoside in Yarrowia lipolytica with yields over 6,400-fold higher than that extracted from its natural plant source.

## Introduction

*Curculigo orchioides* Gaertn (called Xianmao in Chinese), a perennial herb in the family Amaryllidaceae [1–3], was first described as relieving mental fatigue in “Extrinsic Materia Medica” by Li Xun, a Persian doctor in the Tang Dynasty [4]. Since then, it has been used in many formulas of Traditional Chinese Medicine, such as the Immortal Elixir mentioned in “Longevity and Life Preservation” in the Ming Dynasty, or recently in the Erxian decoction in the “Chinese Pharmacopoeia” in the 1950s [5]. A few modern studies have suggested that *C*. *orchioides* has various effects such as anti-inflammatory, antioxidant, anti-osteoporosis, antidepressant, immune regulation, as well as a protective effects on vascular endothelial cells [6–10].

**Fig 1:**
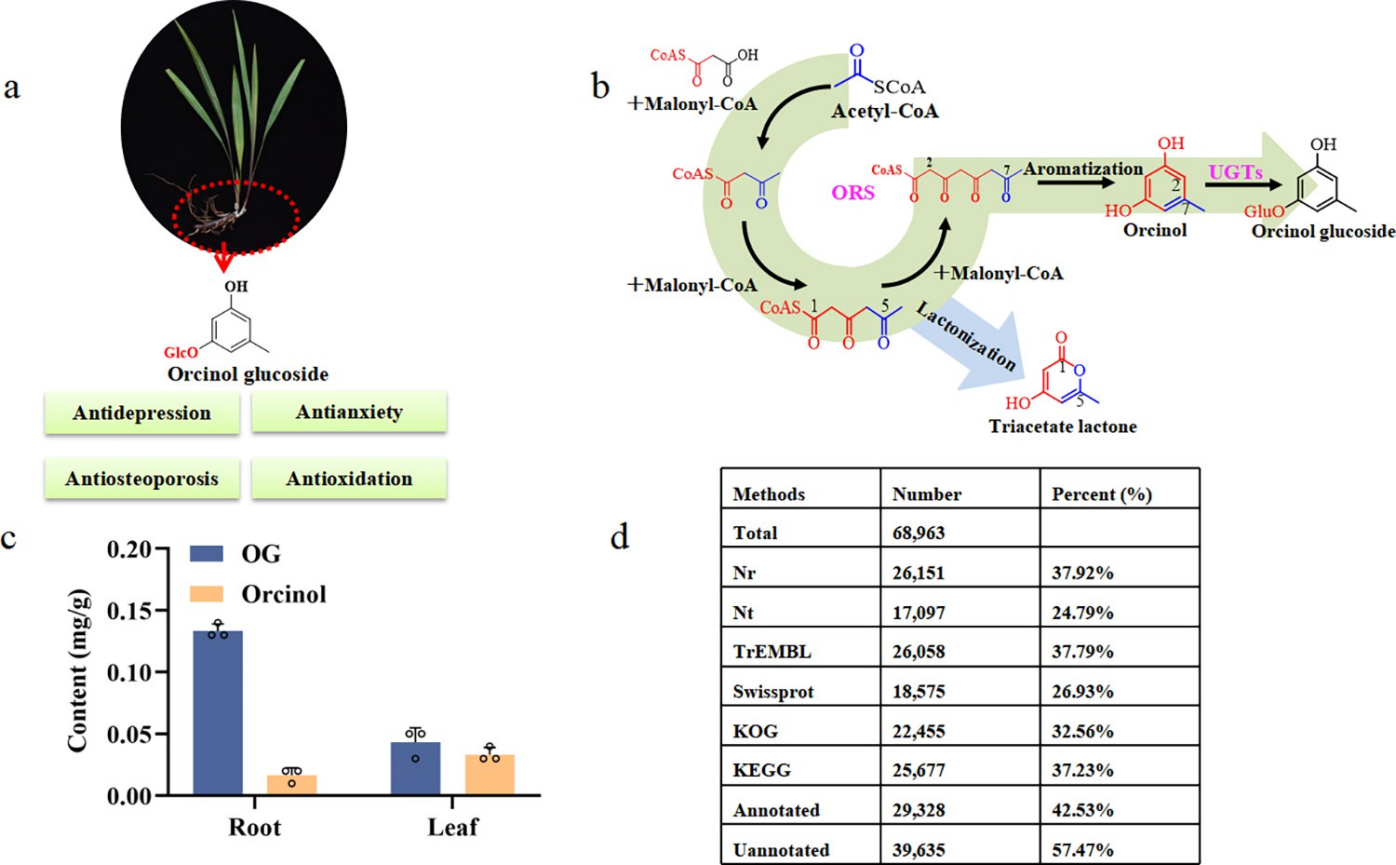
Analysis of the OG biosynthesis pathway. (**a**) Plant and pharmacological effects of *C*. *orchioides*. (**b**) Metabolic pathway analysis of OG. ORS, orcinol synthase; UGTs, UDP-glycosyltransferases. (**c**) Content of orcinol and OG in the roots and leaves of *C*. *orchioides*. All data represent the means from *n* = 3 biologically independent samples and error bars indicate the standard deviations. The circles represent the raw data and listed in the S1 Data. (**d**) Functional annotation of unigenes obtained from *C*. *orchioides*.

Orcinol glucoside (OG) is the major active ingredient of *C*. *orchioides* [11,12], which has been testified to exhibit diverse pharmacological activities, and a few studies have suggested that OG may be the main active component with antidepressant activity in *C*. *orchoides* (Fig 1A) [8,9]. However, the commercial application of OG remained limited due to inefficient plant extraction as well as complex and expensive chemical synthesis [2,13]. Considering that more than 350 million people in the world are currently suffering from depression, OG has been receiving increasing attention due to its antidepressant effect and was investigated in clinical studies as a class I new drug in China [14–16]. In order to establish a green and sustainable production approach, we proposed to de novo biosynthesize OG by engineering the nonconventional oleaginous yeast *Yarrowia lipolytica*, which is an emerging industrial host for the production of various natural products derived from acetyl-CoA and malonyl-CoA [17–19].

Based on transcriptome sequencing of *C*. *orchioides*, computational analysis, and in vitro functional validation, we successfully identified the highly efficient orcinol synthase (ORS) and UDP-dependent glycosyltransferase (UGT) from the OG biosynthesis pathway. De novo biosynthesis of OG was firstly realized in *Y*. *lipolytica* and was then improved approximately 100-fold to 43.46 g/L by optimizing metabolic pathways and fermentation conditions. Our results provide an alternative approach for high-level OG production on an industrial scale.

## Results

### Transcriptome analysis of *C*. *orchioides*

The identification of key enzymes is the cornerstone of the heterologous biosynthesis of plant natural products. We speculated that the biosynthesis of OG is firstly catalyzed by ORS, which is a type III polyketide synthase (PKS) [20], via condensation of 1 molecule of acetyl-CoA and 3 molecules of malonyl-CoA to generate orcinol, which is subsequently modified with UGT at the carbon-3 or carbon-5 hydroxy group to generate OG (Fig 1B). Metabolic analysis and comparative transcriptome analysis were employed to explore the functional genes encoding ORS and UGT in *C*. *orchioides*. The distributions of OG and its precursor orcinol in the roots and leaves of *C*. *orchioides* was evaluated, and the results showed that OG was mainly enriched in the roots, with almost 3-fold greater abundance than in leaves (Fig 1C).At the same time, the total content of OG and orcinol in roots was also higher than in leaves (Fig 1C). Therefore, it was possible to identify the candidates of ORS and UGT by focusing on genes with high expression in roots.

Young leaves and roots of *C*. *orchioides* from Yunnan province of China were used to extract the total RNA. After transcriptome sequencing, a total of 45.54 Gb raw data were obtained, including 42.90 Gb of clean reads, 68,963 unigenes (S1 Fig and S1 Text), and 29,328 annotated proteins, which were identified by searching against the Nr, Nt, TrEMBL, SwissProt, KOG, and KEGG databases (Fig 1D). The genes of *C*. *orchioides* were functionally annotated using GO and KEGG (S2 and S3 Figs and S1 Text). Four candidate sequences were annotated as encoding type III PKS, including 1 chalcone synthase (CHS) and 3 ORS genes (S4 Fig). In addition, 71 candidate sequences of UGTs were obtained (S5 Fig), which provided a basis for subsequent functional screening.

### Characterization of the recombinant ORSs

**Fig 2:**
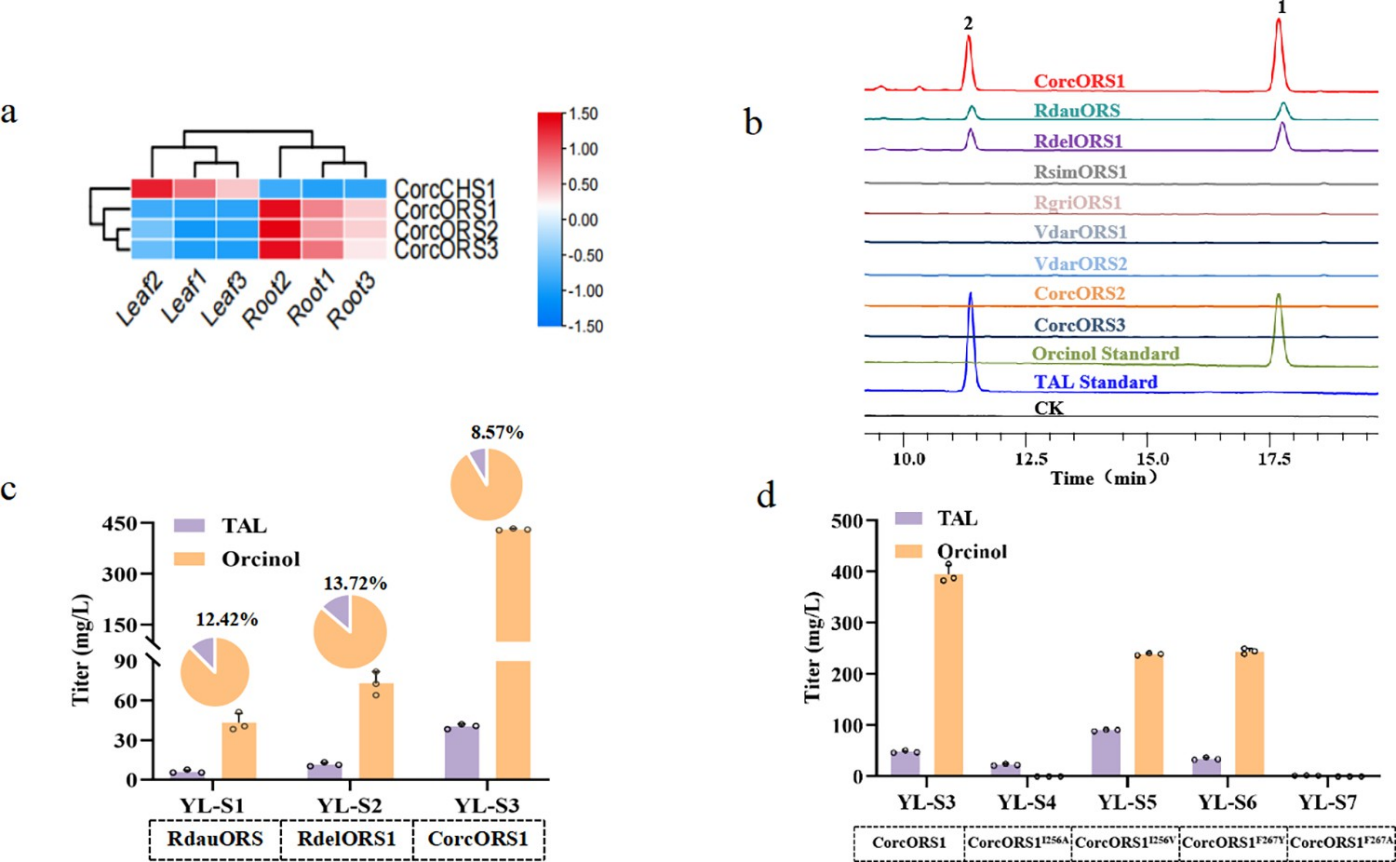
Functional identification of ORS. (**a**) Heatmap analysis of PKS gene expression level in *C*. *orchioides*. (**b**) HPLC analysis of the orcinol standard, TAL standard, and reaction products of different recombinant candidate ORS generated in the presence of acetyl-CoA and malonyl-CoA. Peak1: Orcinol; Peak2: TAL; CK: the control reaction with heat-denatured ORS. (**c**) Orcinol and TAL production catalyzed by different ORSs in shake flask fermentation. All data represent the means of *n* = 3 biologically independent samples and error bars show the standard deviations. The circles represent the raw data and the raw data was listed in S1 Data. (**d**) Orcinol and TAL produced by engineered strains YL-S3 to YL-S7 in shake flask fermentation. All data represent the means of *n* = 3 biologically independent samples and error bars show the standard deviations. The circles represent the raw data and the raw data was listed in S1 Data. ORS, orcinol synthase; PKS, polyketide synthase; TAL, triacetic acid lactone.

According to transcriptomic analysis, all 3 CorcORSs had higher expression levels in roots than in leaves, but the CHS had much higher expression level in leaves (Fig 2A). In addition, we also discovered 5 ORS candidates from other species based on sequences similarity (S4 Fig). All of the ORS candidate genes were characterized using in vitro enzyme activity assays with acetyl-CoA and malonyl-CoA as substrates. We observed 2 peaks produced by CorcORS1, RdauORS, and RdelORS1 (Fig 2B), whereby peak 1 was identical to the orcinol standard and the [M-H]^-^ molecular weight of this new compound was further confirmed by LC-MS (S6 Fig). In addition, we also detected the by-product triacetic acid lactone (TAL) in the reaction system, which was produced by the condensation of 1 molecule of acetyl-CoA with 2 molecules of malonyl-CoA (Figs 2B and S6).

We further confirmed the activities of the orcinol biosynthesis genes in *Y*. *lipolytica* strain W29. Based on the marker-free CRISPR/Cas9 integration system that exploits a highly efficient gRNA to guide Cas9 protein to specific integration sites and knock-in the gene expression cassettes, we constructed orcinol production strains by integrating RdauORS, RdelORS1, and CorcORS1 into the KU80 site of YL0, resulting in the engineered strains YL-S1, YL-S2, and YL-S3. The strain harboring CorcORS1 (YL-S3) showed the highest productivity of orcinol with a titer of 431.06 mg/L, which was 9.93 times higher than that of the reported RdauORS (YL-S1, 43.43 mg/L) and 5.9 higher than that of RdelORS1 (YL-S2, 73.02 mg/L) (Fig 2C). Furthermore, the proportion of the by-product TAL was also the lowest in the strain expressing CorcORS1 (8.57%).

In order to investigate the mechanism underlying the high activity of CorcORS1, we compared the residues in the catalytic pockets of all the studied ORSs. We found that the I256 and F267 residues of CorcORS1 were substituted with Val and Tyr in both RdauORS and RdelORS1 (S7 Fig). Mutations at both positions greatly decreased the activity of CorcORS1 (Fig 2D). Based on the predicted structure, these 2 sites were located in the initiation region and close to the boundary of the elongation region (S8 Fig). Considering that ester condensation reactions require a relatively hydrophobic environment [20], we inferred that these 2 sites might interfere with the hydrophobic nature of the elongation region.

### Characterization of the recombinant UGTs

**Fig 3:**
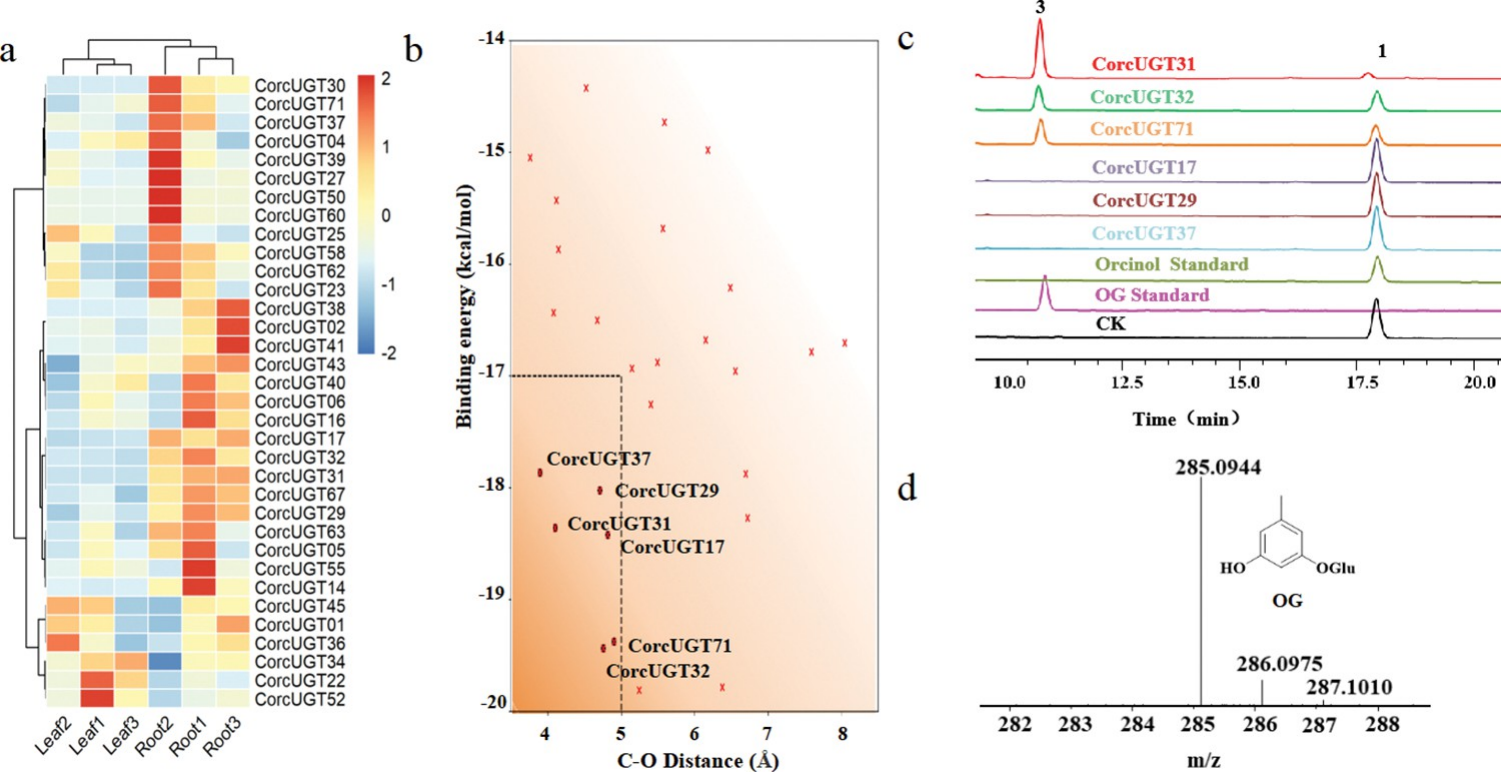
Transcriptome analysis and enzyme activity assay of CorcUGT. (**a**) Heatmap analysis of UGT gene expression levels in *C*. *orchioides*. (**b**) Scatterplot of the substrate binding energy fraction versus the C-O distance for representative docked conformations. The candidate area is delineated by a dotted line. (**c**) HPLC analysis of orcinol standard, OG standard, and reaction products generated by the recombinant CorcUGTs using orcinol and UDP-glucose as precursors. Peak1: Orcinol; Peak3: OG. (**d**) MS analysis of OG in the reaction products of CorcUGT31. OG, orcinol glucoside; UGT, UDP-dependent glycosyltransferase.

The UGT responsible for OG formation in *C*. *orchioides* remains unknown. In order to improve the screening efficiency, a screening pipeline was established in this study for identifying functional UGTs, including transcript level screening, molecular docking, and in vitro enzyme activity assays. Firstly, we analyzed the expression levels of all candidates UGT in roots and leaves. A total of 34 candidate genes with higher expression levels in the roots were used for the subsequent screening (Fig 3A and S5 Table). Secondly, considering the reaction mechanism of UGT, where the hydroxyl group of the substrate is initially deprotonated by an Asp-His pair, and is then glycosylated by a nucleophilic attack on UDP [21,22]. The Asp-His and His-orcinol distances were constrained during the docking screening process, so as to limit the docking results to a near-reactive state (S9 Fig). In this state, the distance between the glycosylated O atom of the substrate and the C1 of the sugar group (C-O distance) is critical for the reaction, and the binding energy of the substrate reflects its stability (S9 Fig). Therefore, the C-O distance and the binding energy of UGT and substrate were used as criteria to screen candidate genes during the docking process (Fig 3B). As a result, 6 candidate genes (CorcUGT17, CorcUGT29, CorcUGT31, CorcUGT32, CorcUGT37, and CorcUGT71) were selected for further functional screening (Fig 3B).

Finally, in vitro enzyme activity assays were conducted to validate these 6 candidate glycosyltransferases. The results showed that 3 UGTs (CorcUGT31, CorcUGT32, and CorcUGT71) produced new peaks with orcinol as substrate, and their HPLC retention times were consistent with the OG standard (Fig 3C, peak 3). Among them, CorcUGT31 had the highest activity and converted 96% of orcinol into OG. LC-MS analysis further confirmed that the [M-H]^-^ molecular weight of this new product generated by CorcUGT31 was the same as that of the OG standard (Fig 3D). Thus, CorcUGT31 was selected for further study.

### Metabolic engineering of OG producing strains

**Fig 4:**
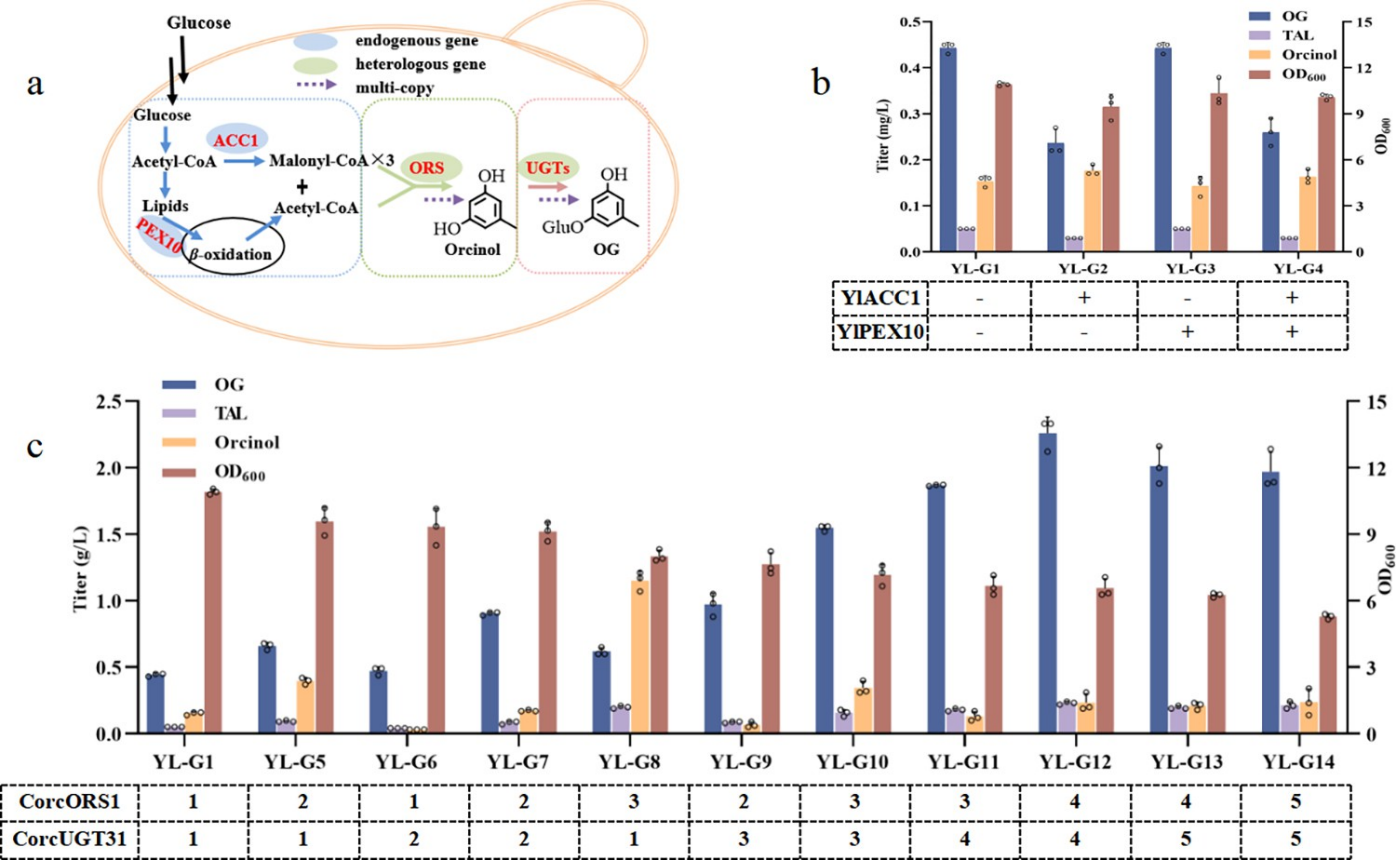
De novo biosynthesis of OG in *Y*. *lipolytica*. (**a**) Metabolic engineering of the OG producing strain. (**b**) Evaluation of OG, TAL, and orcinol produced by engineered strains YL-G1 to YL-G4 in shake flask fermentation. All data represent the means of *n* = 3 biologically independent samples and error bars show the standard deviations. The circle represents the raw data and the raw data was listed in S1 Data. (**c**) Effect of the integration of additional copies of the heterologous pathway on the product titer. Digits show the number of integrated additional copies of OG biosynthetic genes. All data represent the means of *n* = 3 biologically independent samples and error bars show the standard deviations. The circles represent the raw data and the raw data was listed S1 Data. OG, orcinol glucoside; TAL, triacetic acid lactone.

An engineered *Y*. *lipolytica* cell factory was constructed to produce OG in vivo (Fig 4A). The codon-optimized sequences of CorcORS1 and CorcUGT31 were integrated into the genome of *Y*. *lipolytica*, resulting in strain YL-G1. After 4 days of shake flask fermentation, de novo biosynthesis of OG was realized in *Y*. *lipolytica* for the first time (S10 Fig), but the product titer was only 0.44 g/L (Fig 4B). By analyzing the proportions of extracellular fermentation products of strain YL-G1, we found that the products were mostly transported out of the cells. The extracellular ratios of OG, TAL, and orcinol were 95.78%, 96.42%, and 92.10%, respectively (S11 Fig and S2 Text). Thus, the products were almost completely transported outside of the cell, which was beneficial for the scale-up of fermentation and purification of target products.

Previous studies showed that overexpression of peroxisomal biogenesis factor 10 (PEX10) could divert acetyl-CoA from lipid synthesis into naringenin or TAL [23,24], while overexpression of native acetyl-CoA carboxylase 1 (ACC1) could promote the conversion of acetyl-CoA into malonyl-CoA [25] (Fig 4A). To investigate whether the precursor supply of acetyl-CoA and malonyl-CoA limited the synthesis of OG, the endogenous YlPEX10 and YlACC1 genes were overexpressed in YL-G1, respectively (Fig 4A). However, the production of OG was not improved at all (Fig 4B), which might be caused by an imbalance in the synthesis of long-chain fatty acids and impaired cell growth [26–28].

The metabolic flux towards the downstream pathway was enhanced by multicopy integration of key genes. When only the copy number of CorcORS1 was increased, the accumulation of orcinol in the corresponding strains YL-G5 (0.4 g/L), and YL-G8 (1.15 g/L) was 2.67 and 7.67 times higher than in YL-G1, respectively (Fig 4C). When the copy number of CorcUGT31 was increased, the transformation from orcinol to OG was more complete in strains YL-G6 and YL-G9, with residual orcinol titers of 0.03 g/L and 0.07 g/L. In order to identify the optimal relative gene dosage of CorcORS1 and CorcUGT31, multiple rounds of integration were performed using the same marker-free CRISPR/Cas9 integration system, which enabled accurate and predictable chromosomal editing without multiple tag recovery [29]. The yield of OG in strain YL-G12 carrying 4 additional copies of CorcORS1 and CorcUGT31 was increased to 2.23 g/L, which was 5.14 times higher than in YL-G1 (Fig 4C). However, when a fifth additional copy of CorcORS1 and CorcUGT31 was added, the yield slightly decreased to 1.97 g/L, and the OD_600_ also decreased to 5.29 (Fig 4C). We firstly speculated that excess accumulation of OG may result in cytotoxicity. Therefore, the OD_600_ of wild-type W29 was measured after 96 h of growth with the external addition of OG, but there was no significant decrease of OD_600_ at any of the tested OG concentrations (S12 Fig). Thus, we speculated that the reason for decreased cell growth might be the metabolic burden imposed by multiple copies of heterologous genes. Ultimately, the best strain YL-G12 was used for further experiments.

### High-level production of OG in fed-batch fermentation

**Fig 5:**
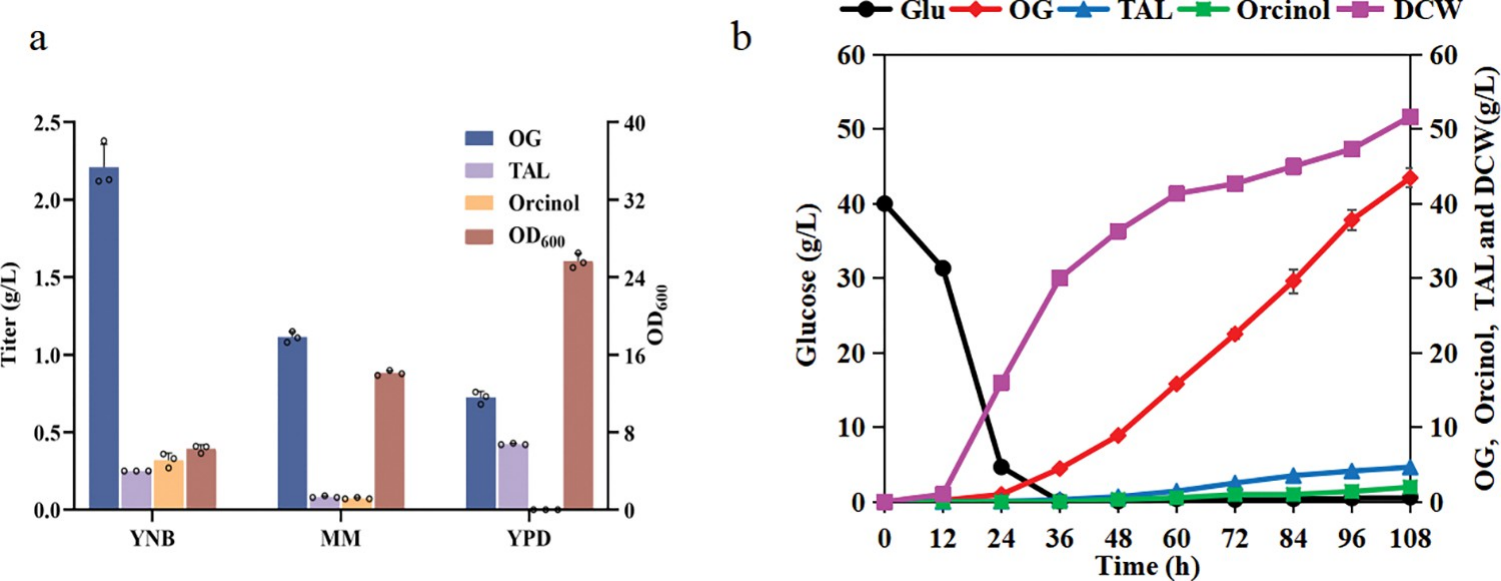
Fed-batch fermentation of the engineering strain YL-G12 in a 1.3-L bioreactor. (**a**) Shake flask fermentation of engineered strain YL-G12 in different media. All data represent the means of *n* = 3 biologically independent samples and error bars show the standard deviations. The circles represent the raw data and the raw data was listed in S1 Data. (**b**) OG, TAL, and orcinol production of engineered strain YL-G12 in fed-batch fermentation and the raw data was listed in S1 Data. OG, orcinol glucoside; TAL, triacetic acid lactone.

The medium has been proven to be one of the most important factors for high-yield chemical production in *Y*. *lipolytica* [19,30]. Three different media were compared in the fermentation process of the engineered strain YL-G12. Among them, the strain exhibited the highest product titer in YNB medium and the highest growth rate in YPD medium. Although the growth and production in MM medium were at a moderate level, the by-product titer of TAL was only 0.09 g/L, corresponding to the lowest observed ratio of 6.76% (Fig 5A). Considering that YNB is more expensive and has a higher tendency to generate foam than MM medium, we finally selected MM medium to carry out fed-batch fermentation in a bioreactor.

In order to further improve the production of OG and test whether *Y*. *lipolytica* could be used as an industrial host for OG production, we conducted a fed-batch fermentation of the best engineered strain YL-G12. The fermentation process was dynamically controlled by adjusting the pH to 5.0 and the dissolved oxygen level to 30% of atmospheric saturation. The rebound of DO after glucose was exhausted was used as a signal to start, feeding at an initial rate of 3 g/h, and the residual glucose concentration was kept below 1 g to maintain cell growth. OG began to accumulate after 24 h and the highest yield reached 43.46 g/L at 108 h, which was almost 100 times higher than the original yield in shake flasks. A total of 174.98 g glucose was consumed over a span of 108 h and the DCW reached 51.67 g/L, with a percent yield of 28.13% and productivity of 0.4 g/L/h (Fig 5B and S3 Text).

Notably, the OG-producing *Y*. *lipolytica* strain YL-G12 formed hyphae during the later stage of fermentation. It has been reported that *Y*. *lipolytica* is a dimorphic species that can undergo a yeast-to-mycelium transition when it is exposed to stress, and the mycelial form is unfavorable for industrial fermentation [31,32]. Thus, we also tested the effect of metabolic stress related to mycelial growth by deleting the CLA4 and MHY1 genes to convert the mycelium back to the yeast form [31–34]. Although we successfully reverted the double-knockout strain YL-G17 to the yeast form (S13 Fig), the production of OG was similar to the parental strain YL-G12 (S14 Fig). Therefore, the morphological change might be not a limiting factor for the production of OG under the conditions applied in this study.

## Discussion

Development of enabling technology to facilitate the gene mining process of catalytic elements with high activities would accelerate the construction of microbial cell factories for the production of valuable natural products. Considering that the traditional Chinese herb *C*. *orchioides* was rich in the antidepressant orcinol glucoside, we explored its functional genes for the biosynthesis of OG. By integrating transcriptome sequencing, constrained molecular docking, and functional characterization, we constructed a pipeline to narrow down the candidate genes encoding UGTs. The restrictions of gene expression and substrate binding rapidly eliminated more than 90% of potential candidates. The labor-intensive functional validation of candidate UGTs was then conducted on only 6 genes, avoiding tedious experimental testing. This procedure provides a strategy for the discovery of new glycosyltransferases and other functional genes in the biosynthetic pathways of plant natural products, which has also been applied to construct the comprehensive plant UGT database pUGTdb (http://pugtdb.biodesign.ac.cn/).

Improvement of cell factory productivity is a complex proposition. We comprehensively considered host cells, metabolic engineering, medium, and fermentation conditions to improve the microbial production of OG. The emerging industrial microorganism *Y*. *lipolytica* is naturally endowed with high carbon fluxes towards acetyl-CoA and malonyl-CoA [17,18]. For example, the overexpression of the endogenous YlPEX10 and YlACC1 genes, which had been used to improve the supply of acetyl-CoA and malonyl-CoA [23,24], did not improve the OG production, indicating that the precursor supply was not the limiting factor. Indeed, the overexpression of downstream genes (CorcORS1 and CorcUGT31) in the biosynthetic pathway of OG greatly improved the production of OG approximately 5.14-fold. At the same time, we found that the choice of a suitable fermentation medium not only largely determined the productivity, but was also critical for reducing by-product synthesis. After additional optimization of fed-batch fermentation conditions, the productivity of OG in the engineered strain YL-G12 was improved approximately 100-fold compared to the starting strain YL-G1. However, at present the calculated percent yield of OG from glucose in YL-G12 is only 28.13%. It is worth mentioning that the cell growth consumes about 68.2 g of glucose and the percent yield is 46.11% when not considering the glucose consumed by cell growth (S3 Text). This suggests that there is still plenty of space to improve the conversion rate by metabolic and fermentative optimization, although the yield of OG in the engineered strain has reached the requirements of industrial production.

Notably, the engineered yeast developed in this study reached an OG productivity value of 0.84 g/g DCW, which was 6,400-fold higher than the yield of plant extraction from *C*. *orchioides* roots (0.131 mg/g DCW), indicating a remarkable improvement of OG production in yeast through synthetic biology. Currently, the planting cycle of *C*. *orchioides* is 5 years and the yield of plants is about 4,500 kg/ha (approximately 900 kg dry weight). Accordingly, the OG yield of our strain in a 1 cubic meter fermentor during 5 years will be approximately equal to the yield of OG from plants grown on 149,287 ha (details of calculations were added to the S4 Text). In addition to the highly favorable economics of the fermentation compared to planting, it also saves more than 90% of fresh water resources, while also avoiding the negative impact of pesticides and fertilizers on the environment.

Depression has become an epidemic mental illness, affecting approximately 16% of the population [8,35]. Chemical treatments are limited by side effects such as myorelaxation, headache, tachycardia, etc. [8]. However, highly effective and safe therapeutic solutions could be found in traditional Chinese medicine, which has used *C*. *orchioides* to treat depression for centuries. Here, the biosynthesis of OG was realized for the first time by developing an efficient microbial cell factory with a high titer of 43.46 g/L, attesting the potential of *Y*. *lipolytica* for the production of plant secondary metabolites with a malonyl-CoA-derived structure. Our study lays a solid foundation for industrial-scale fermentation of OG in a green and efficient manner, which will greatly benefit translational research on the treatment of depression in the near future.

## Materials and methods

### Reagents, media, and culture conditions

Luria broth (LB), agar, yeast extract, ampicillin, kanamycin, and peptone were obtained from Solarbio, China. The standards of acetyl-CoA, malonyl-CoA, orcinol, OG, and TAL were obtained from Weikeqi, China. *Escherichia coli* cells were cultured at 37°C in LB, supplemented with appropriate antibiotics, such as ampicillin or kanamycin, at a final concentration of 100 mg/L. The wild-type *Y*. *lipolytica* strain W29 (ATCC No. 20460) obtained from the ARS Culture Collection (NRRL), which was used as the starting strain for the construction of all the engineered strains. *Y*. *lipolytica* strains were grown in YNB or YPD medium at 30°C and 220 rpm. The gRNA plasmid pCfB6627 was obtained from Addgene (https://www.addgene.org/), article number #106159. Composition of media are listed in S5 Text.

### Extraction of OG and orcinol from plant tissues

The plant material of *C*. *orchioides* was collected from Shidian County (25° 08′ N, 99° 10′ E), Baoshan City, Yunnan Province, China. To extract OG and orcinol, the dried roots and leaves were separately ground into a powder, 1 g of which was extracted in 50 mL of ethanol, as described previously [36].

### RNA extraction, transcriptome assembly, and gene annotation

This experimental library was established by Guangzhou Gene Denovo Biotechnology Co. Total RNA was extracted from young leaves and roots of *C*. *orchioides* using a Trizol RNA Extraction Kit (Promega, United States of America). The RNA samples were then used for cDNA synthesis. Trinity (version 2.13.2) was used to transcriptome assembly (parameters: k-mer 31, min_kmer_cov 2). RapClust (version 0.1.2) was used to obtain final unigenes with remove redundant by clustering. TransDecoder v5.5.0 software was used to predict the coding sequences (CDS) of unigenes. BLAST v2.12.0 (with a threshold E-value of 1 × 10^−5^) was used to compare the predicted CDS with the local KEGG, KOG, Nr, Nt, TrEMBL, and SwissProt databases, and the sequence with the best alignment was selected as the final annotation sequence. Pfam_scan v1.6 was used to annotate the *C*. *orchioides* protein sequences based on the Pfam database (version35.0), E-value < 1 × 10^−5^ as candidate sequence, putative PKS proteins (Pfam ID: PF02797 and PF00195) were screened by amino acid length (length >280). UGTs proteins (Pfam ID: PF00201) were screened by amino acid length (length >250). InterproScan v5.55–88.0 was used to annotate to GO; kofam_scan v1.3.0 was used to annotate sequences to KO. Venn diagrams were drawn using Btools (0987 version).

### Sequence alignment, phylogenetic analysis, and heatmaps

The multiple sequence alignments of proteins were generated using MAFFT v7.505 (parameters:—maxiterate 1000—localpair), and the results were pruned using trimAl v1.4.1 (parameters: -gt 0.02). *Arabidopsis thaliana* genome data were downloaded from an online database (http://www.p450.kvl.dk/).The reference sequence was downloaded from the NCBI database (http://www.ncbi.nlh.gov/). The phylogenetic NJ tree analysis of plant type III PKSs was built using phylip v3.698, bootstrap was seedt 1000, the phylogenetic ML tree analysis of UGT was built using IQ-TREE v2.1.4-beta with a model of LG+F+R10 and parameter of -B 1000 -alrt 1000. The phylogenetic tree was rendered using EvolView (https://evolgenius.info/evolview/). TBtools v1.098722 was used to draw the heatmap of candidate genes.

### Construction of plasmids and strains

All primers, plasmids, strains, and genes used in this study relisted in S1 to S4 Tables, respectively. All primers were synthesized by Tsingke Biotechnology Co. (China). All codon-optimized heterologous genes were synthesized by GenScript (China). The amplified ORS coding genes were purified and subcloned into the pQE-80L vector digested with *Bam*HI and *Sal*I, using the super fusion cloning mix (US Everbright). The resulting constructs, which direct the synthesis of the recombinant proteins with an N-terminal hexahistidine (6×His) tag, were introduced into *E*. *coli* M15. The UGT genes were digested and ligated into the expression vector pET-28a between the *Bam*HI and *Xho*I sites, and the resulted plasmids were respectively introduced into *E*. *coli* BL21 (*DE3*).

To construct integrative strains of *Y*. *lipolytica*, a single gRNA plasmid and a linearized homologous donor plasmid were introduced into the cells via the lithium acetate transformation method the same as ref [37,38], which resulted in chromosomal insertion via the CRISPR/Cas9 system. For application of CRISPR/Cas9 system, the Cas9 expression cassette was integrated into the CDS region of KU70. Briefly, the Cas9 cassette, HygR cassette, and two 2,000-bp homologous arms were fused and amplified by overlap-PCR, and then the fragment was transformed into *Y*. *lipolytica* W29 cells. The positive colonies were selected on YPD plates with hygromycin B and checked by colony-PCR. The primers were provided in S1 Table. The integrative plasmid was constructed using pMD-19T as the backbone with different integration sites, such as KU80, IntC-2, IntC-3, IntD-1, IntE-1, and IntE-3; and the integration efficiency of these sites is above 80% [38]. Optimal target-specific sgRNA sequences (20 bp) were identified with the help of the online tool “CHOPCHOP” (http://chopchop.cbu.uib.no/) [38]. The single-gRNA plasmid was prepared as described previously [37]. Relevant details of the construction of plasmids and related fragments are listed in S6 Text. Relevant details of the flask fermentation of *Y*. *lipolytica* strains are listed in S7 Text.

### Bacterial expression and purification of the recombinant ORS and UGT enzymes

The recombinant *E*. *coli* cells were cultured in a 2 L shaker containing 800 mL of LB medium (LB medium containing 100 mg/L ampicillin or kanamycin), no baffles added, at 37°C, 220 rpm until the OD_600_ reached 0.6 to 0.8, after which expression was induced by adding isopropyl *β*-D-thiogalactoside (IPTG) at a final concentration of 0.1 mM and continued at 16°C for 14 h. Then, the cells were harvested by centrifugation at 5,000 g for 20 min, resuspended in 30 mL of buffer A [50 mM Tris-HCl (pH 8.0) containing 200 mM NaCl], the high-pressure homogenizer 100 (JNBIO, 1,200 bar) cracked the bacteria to release proteins. The homogenate was centrifuged at 10,000 g for 40 min to remove cell debris, and the cleared supernatant was applied to a Ni Sepharose 6 Fast Flow column (1.0 cm × 2.5 cm) equilibrated with buffer A. After the sample was applied to the column, nonspecifically bound proteins were removed using buffer A containing 20 mM, 40 mM, 60 mM, 80 mM, and 100 mM imidazole, sequentially. The 6×His-tagged recombinant proteins were then eluted with buffer A containing 200 mM imidazole. The purity and subunit molecular masses of the recombinant ORS and UGT were verified by SDS-PAGE analysis (S15 Fig), and the protein concentration was determined using a BCA Protein Assay Kit (Pierce, USA) with 2 mg/mL BSA as the standard. Purified enzymes were stored at −80°C before use.

### Enzyme activity assays

The standard reaction mixture consisted of 1 mM acetyl-CoA, 2 mM malonyl-CoA, and 3 mg of the purified recombinant ORS in buffer A, and the total volume was 100 μL. The CorcUGT reactions were conducted in a similar manner, with 100 μL reaction mixtures containing 1 mM orcinol (sugar acceptor), 2 mM UDP-Glucose (sugar donor), and 3 mg of the purified recombinant CorcUGT in buffer A. The control reaction was conducted in the same reaction system with heat-denatured purified CorcORS or CorcUGT. After incubating the reaction mixture for 12 h at 30°C, the reaction was stopped by adding 100 μL of methanol.

### HPLC and LC-MS analysis

The reaction mixture or fermentation broth was centrifuged at 12,000 g for 15 min to remove insoluble components and subjected to HPLC analysis on an Agilent 1260 HPLC system equipped with a ZORBAX SB-C18 column (4.6 × 250 mm, 5-Micron, made in USA). Solvent A was an aqueous solution of 0.1% formic acid and solvent B was acetonitrile. The temperature of the column oven was set to 30°C and 10 μL of the sample was injected at a flow rate of 1 mL/min. The gradient program was as follows: 0 to 3 min, 7% solvent B; 3 to 17 min, solvent B increased from 7% to 17%; 17 to 22 min, solvent B increased from 17% to 90%; 22 to 25 min, solvent B kept at 90%; 25 to 30 min solvent B decreased from 90% to 7%. The products were detected by measuring the absorption at 275 nm.

For LC-MS analysis, samples were analyzed on an Agilent 1260 HPLC system coupled with a Bruker MicrOTOF-Q II mass spectrometer (Bruker, Germany) equipped with an electrospray ionization (ESI) source. All spectra were recorded in negative ion mode over an m/z range of 50 to 500 under dry N_2_ gas flow at 6.01 L min^−1^, a temperature of 180°C, a nebulizer pressure of 1 bar, and probe voltage of 4.5 kV.

### Fed-batch fermentation

Cryopreserved cells from glycerol stock were streaked onto an YPD agar plate and grown for 2 days at 30°C. Then, single colony of *Y*. *lipolytica* was transferred from the plate into 4 mL of MM medium and grown for 48 h at 30°C and 220 rpm. The resulting seed culture was used to inoculate 40 mL of MM medium and grown for 1 day. Fed-batch cultivations were performed in a 1.3 L bioreactor with an initial working volume of 400 mL. The temperature was maintained at 30°C throughout the fermentation. The pH was maintained at 5.0 by the automatic addition of 4 M KOH. The dissolved oxygen (DO) was controlled at 30% of atmospheric saturation via automatic control of the agitation speed (200 to 1,200 rpm), and the initial aeration rate was set to 0.5 L/min. After the initial glucose was exhausted, the feed solution was added to maintain the glucose concentration at approximately 1 g/L. Samples comprising 5 mL of the culture broth were taken every 12 h to analyze the dry cell weight (DCW), residual glucose content, and OG accumulation. All assays were performed at least in triplicate.

### Determination of the biomass and sugar concentration

A sample comprising 1 mL of the fermentation broth was centrifuged at 12,000 g for 2 min to remove the supernatant prior to lyophilization, and DCW was measured after freeze-drying and weighing the cells. The residual glucose concentration was measured using an SBA-40D biosensor (Shandong Academy of Sciences, China).

### Computational analysis

Protein structures of ORSs were predicted using alphafold2. The highly expressed UGTs (FPKM average >10) in the roots were used for the subsequent screening. Docking was performed using the Rosetta package [39] with a distance constraint. The first group constraint (1.5 Å) between the conserved aspartic acid and histidine was the basis of the carbonylation reaction. The second group constraint (1.8 Å) was between the hydroxyl hydrogen of the substrate and the nitrogen of the conserved histidine. These 2 constraints were selected to represent a potential pre-reactive state (S9 Fig), and the binding energy of the substrate was calculated. The binding energy and C-O distance were used as the index to screen candidate genes during the docking process.

## Supporting information

S1 Fig Unigene length distribution. (TIF)

S2 Fig Gene Ontology annotation classification of C. orchioides. (TIF)

S3 Fig KEGG annotation classification of C. orchioides. (TIF)

S4 Fig Phylogenetic tree analysis of plant type III PKSs. The blue asterisk represents known functional gene. The red asterisk represents the candidate gene of *C*. *orchioides*. The green asterisk represents the gene of *Ericaceae Juss*, RdelORS1 from *Rhododendron delavayi*, RgriORS1 from *Rhododendron griersonianum*, RsimORS1 from *Rhododendron simsii*, VdarORS1, and VdarORS2 from *Vaccinium darrowii*.
(TIF)

S5 Fig Phylogenetic tree analysis of UGTs. **A total of** 71 UGT candidate sequences were clustered into 17 subfamilies by using the UGT family of *A*. *thaliana* as the background.
(TIF)

S6 Fig MS analysis of orcinol and TAL in enzymatic reaction systems. (a) MS analysis of orcinol in the reaction products of CorcORS1 (the orcinol standard on the left; the sample of reaction products on the right). (b) MS analysis of TAL in the reaction products of CorcORS1 (the TAL standard on the left; the sample of reaction products on the right).
(TIF)

S7 Fig Sequence analysis in the initiation pocket. Cyclized pocket residues (towards the pocket only).
(TIF)

S8 Fig The different residues of CorcORS and RdauORS in the Initiation pocket. (TIF)

S9 Fig Schematic diagram of the restricted docking screening strategy. The confinement atom pairs are connected by red dashed lines, and the atomic distance of the glycosylated O atom in orcinol and the C1 in UDP-Glucose was used as one of the screening scale and connected by purple dashed lines.
(TIF)

S10 Fig HPLC analysis of the orcinol standard, TAL standard, OG standard, and fermentation products of engineered strains YL-G1 in shake flask. Peak1: Orcinol; Peak2: TAL; Peak3: OG.
(TIF)

S11 Fig Extracellular and intracellular distributions of OG, TAL, and orcinol and the specific data was listed in S1 Data. (TIF)

S12 Fig Effects of different concentrations of OG on W29 strain growth and the specific data was listed in S1 Data. (TIF)

S13 Fig Cell morphology varieties of CLA4 and MHY1 deletion strains. Microscopic images of strains YL-G12, YL-G12△MHY1, YL-G12△CLA4, and YL-G12△MHY1△CLA4.
(TIF)

S14 Fig Shake flask fermentation of engineered strains YL-G12, YL-G15, YL-G16, YL-G17, and the specific data was listed in S1 Data. (TIF)

S15 Fig The 15 SDS-PAGE analysis of the purified recombinant enzymes ORSs and UGTs. M: Marker, 1: CorcORS1, 2: RuauORS, 3: RdelORS1, 4: CorcUGT31, 5: CorcUGT32, 6: CorcUGT71.
(TIF)

S1 Table Primers used in this study. (XLS)

S2 Table Plasmids used in this study. (XLS)

S3 Table Strains used in this study. (XLS)

S4 Table Codon-optimized genes for Y. lipolytica used in this study. (XLS)

S5 Table The FPKM analysis of 71 UGTs from C. orchioides. (XLS)

S1 Data Excel spreadsheet containing the underlying numerical data for Figs 1C, 2C, 2D, 4B, 4C, 5A, 5B, S11, S12 and S14. (XLSX)

S1 Text Methods for plasmids construction. (DOCX)

S2 Text Proportion of OG, orcinol, and TAL inside and outside of cells. (DOCX)

S3 Text Percent yield calculation for the fed batch experiment. (DOCX)

S4 Text “Green and sustainable” calculation. (DOCX)

S5 Text Composition of media. (DOCX)

S6 Text Methods for plasmids construction. (DOCX)

S7 Text Flask fermentation. (DOCX)

S1 Raw Images The raw images for Figs 1A, 2B, 3C, 3D, S6, S10 and S15. (PDF)

## Abbreviations

ACC1: acetyl-CoA carboxylase 1
CHS: chalcone synthase
DCW: dry cell weight
DO: dissolved oxygen
ESI: electrospray ionization
IPTG: isopropyl β-D-thiogalactoside
LB: Luria broth
OG: orcinol glucoside
ORS: orcinol synthase
PEX10: peroxisomal biogenesis factor 10
PKS: polyketide synthase
TAL: triacetic acid lactone
UGT: UDP-dependent glycosyltransferase
