# Supplementary figures and images for: Production of the antidepressant orcinol glucoside in Yarrowia lipolytica with yields over 6,400-fold higher than plant extraction
Source: PLoS Biol. 2023 Jun 6;21(6):e3002131. doi: 10.1371/journal.pbio.3002131 (PMC10243626; doi:10.1371/journal.pbio.3002131)

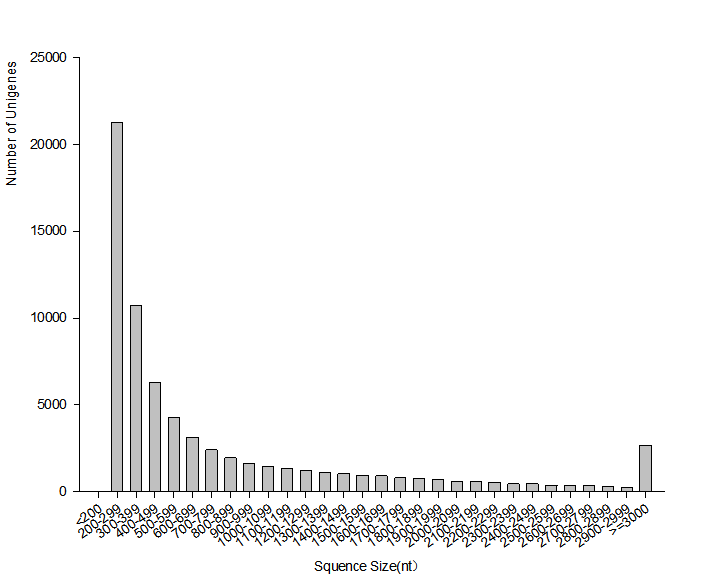

Supplement: S1 Fig — (TIF) [file pbio.3002131.s001.tif]

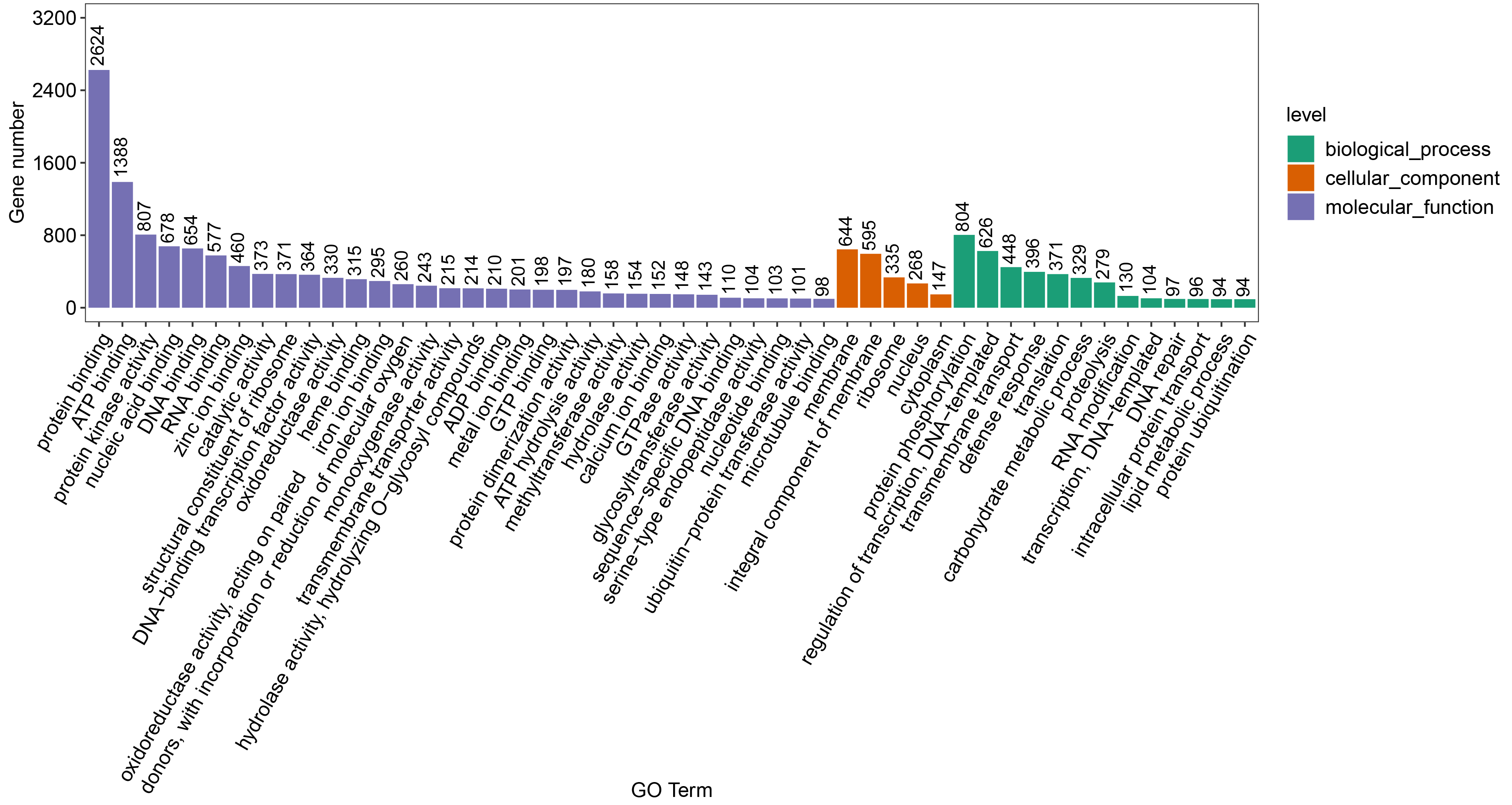

Supplement: S2 Fig — (TIF) [file pbio.3002131.s002.tif]

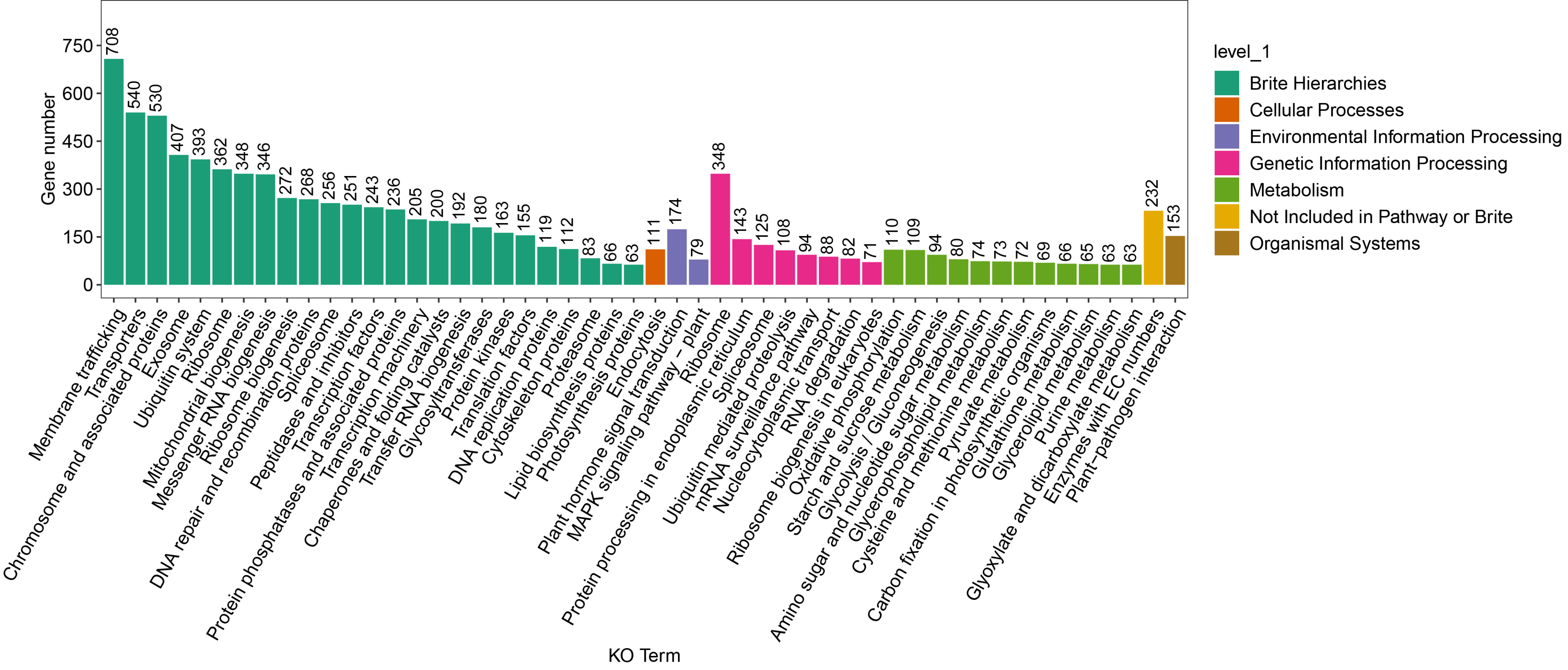

Supplement: S3 Fig — (TIF) [file pbio.3002131.s003.tif]

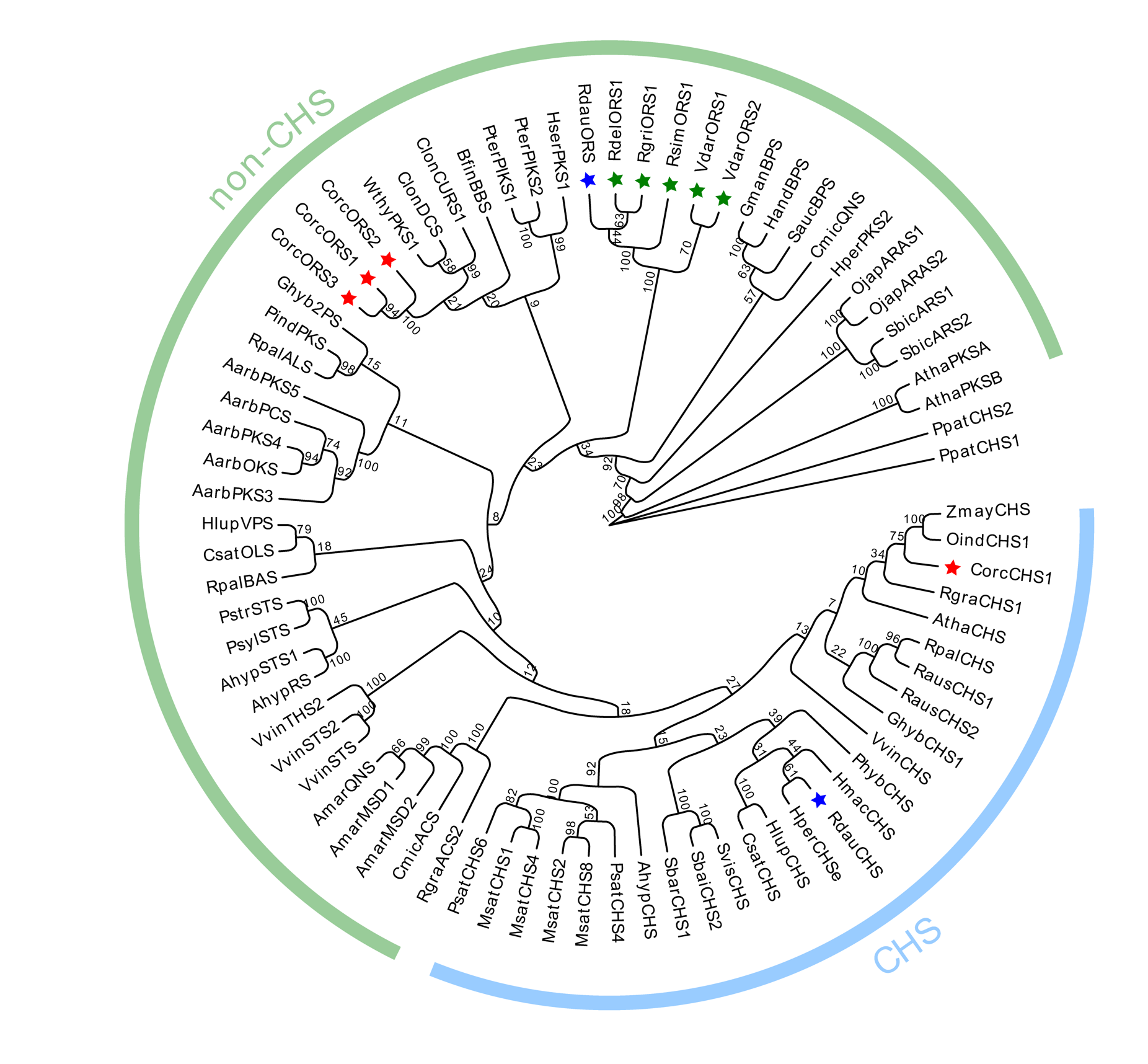

Supplement: S4 Fig — The blue asterisk represents known functional gene. The red asterisk represents the candidate gene of C. orchioides. The green asterisk represents the gene of Ericaceae Juss, RdelORS1 from Rhododendron delavayi, RgriORS1 from Rhododendron griersonianum, RsimORS1 from Rhododendron simsii, VdarORS1, and VdarORS2 from Vaccinium darrowii. (TIF) [file pbio.3002131.s004.tif]

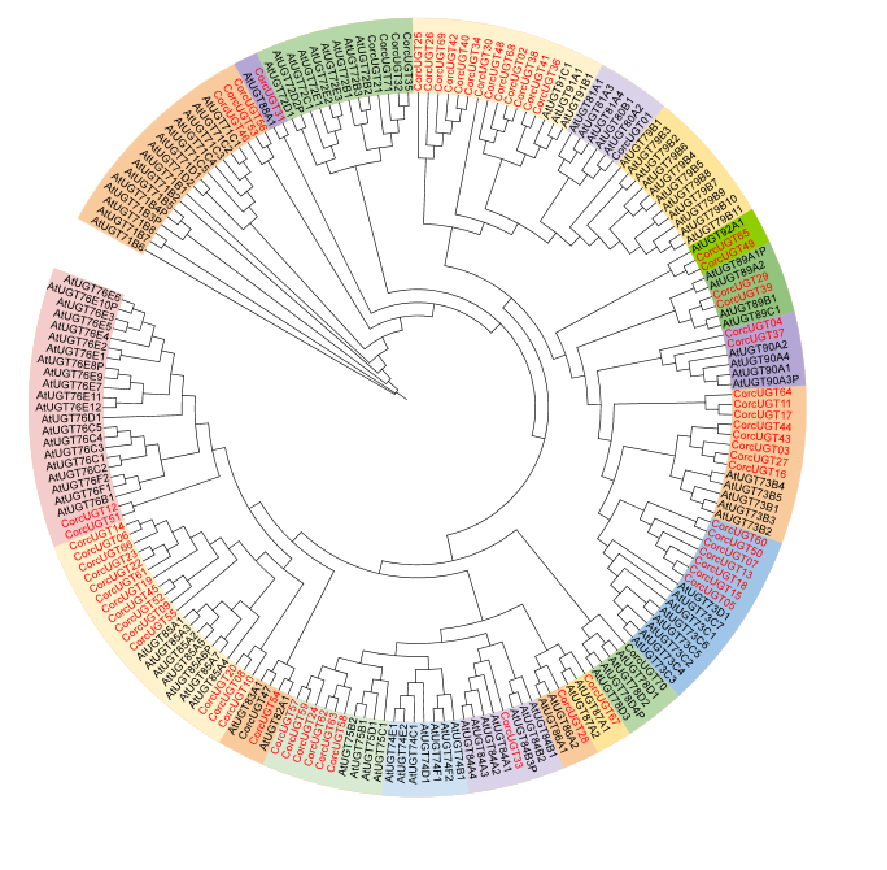

Supplement: S5 Fig — A total of 71 UGT candidate sequences were clustered into 17 subfamilies by using the UGT family of A. thaliana as the background. (TIF) [file pbio.3002131.s005.tif]

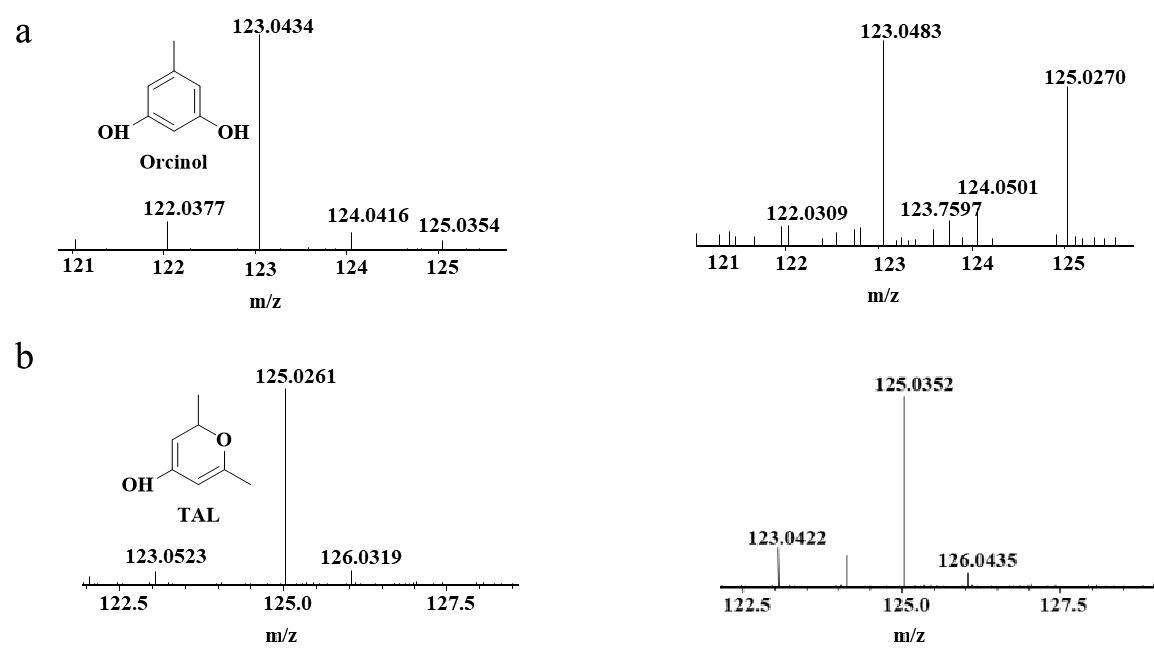

Supplement: S6 Fig — (a) MS analysis of orcinol in the reaction products of CorcORS1 (the orcinol standard on the left; the sample of reaction products on the right). (b) MS analysis of TAL in the reaction products of CorcORS1 (the TAL standard on the left; the sample of reaction products on the right). (TIF) [file pbio.3002131.s006.tif]

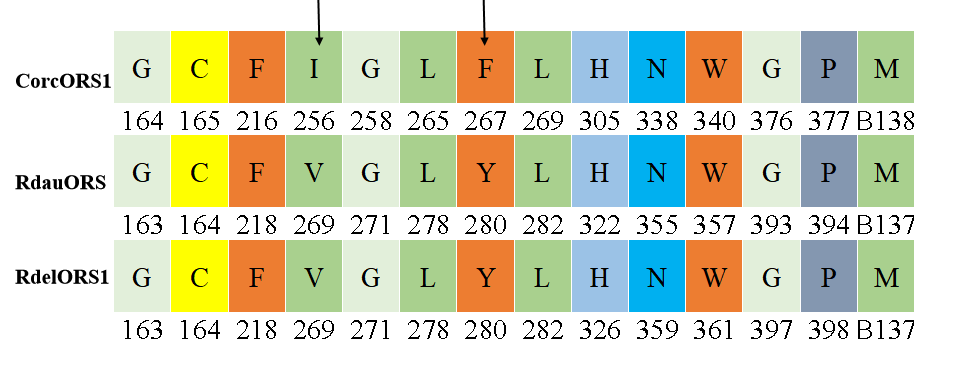

Supplement: S7 Fig — Cyclized pocket residues (towards the pocket only). (TIF) [file pbio.3002131.s007.tif]

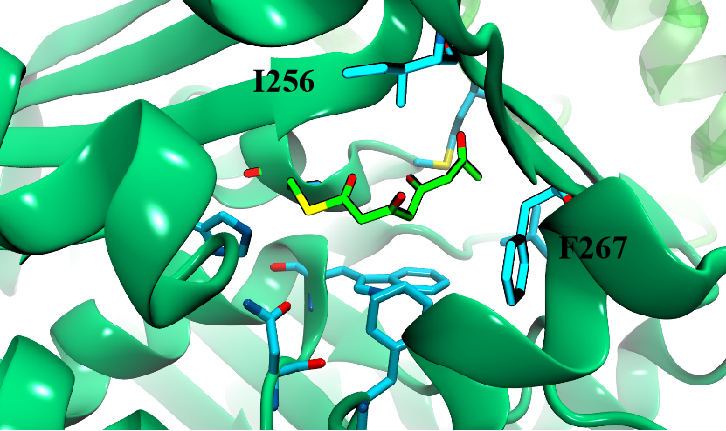

Supplement: S8 Fig — (TIF) [file pbio.3002131.s008.tif]

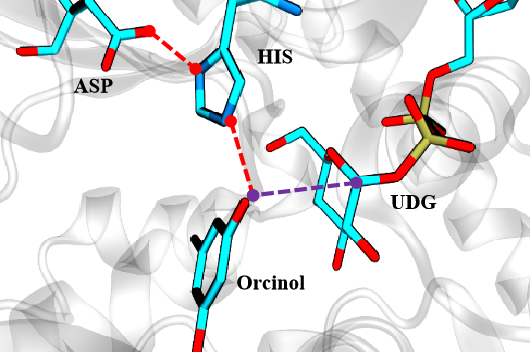

Supplement: S9 Fig — The confinement atom pairs are connected by red dashed lines, and the atomic distance of the glycosylated O atom in orcinol and the C1 in UDP-Glucose was used as one of the screening scale and connected by purple dashed lines. (TIF) [file pbio.3002131.s009.tif]

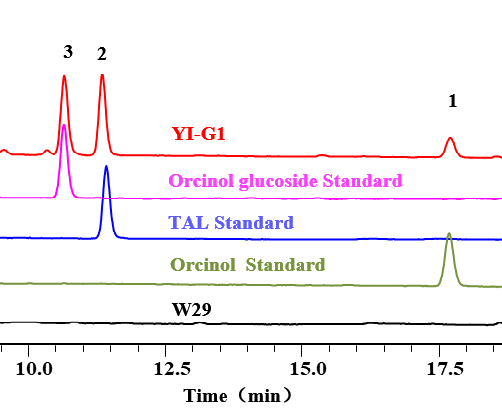

Supplement: S10 Fig — Peak1: Orcinol; Peak2: TAL; Peak3: OG. (TIF) [file pbio.3002131.s010.tif]

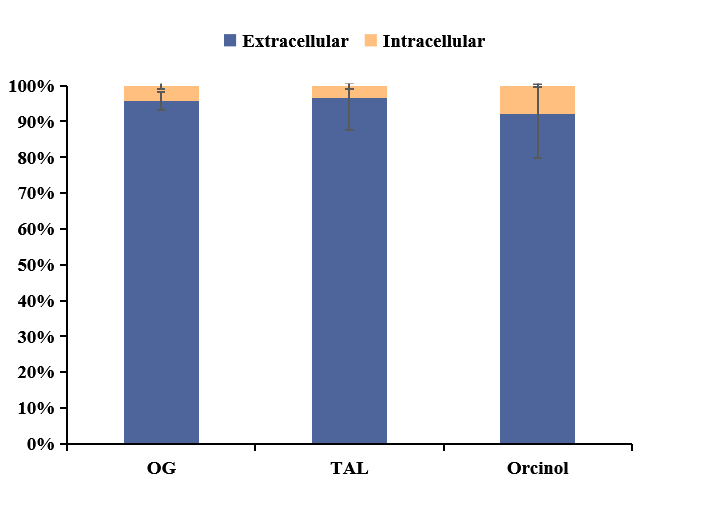

Supplement: S11 Fig — (TIF) [file pbio.3002131.s011.tif]

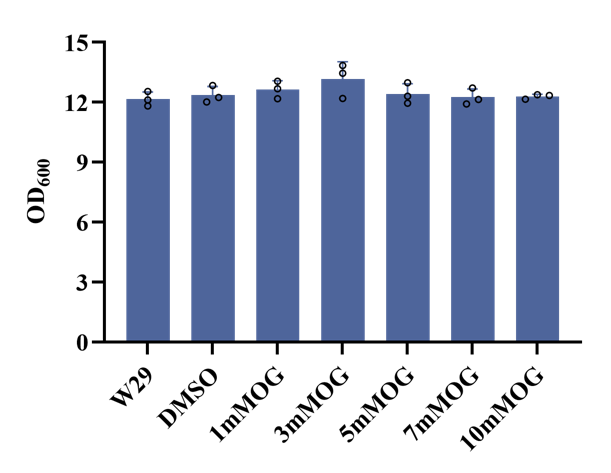

Supplement: S12 Fig — (TIF) [file pbio.3002131.s012.tif]

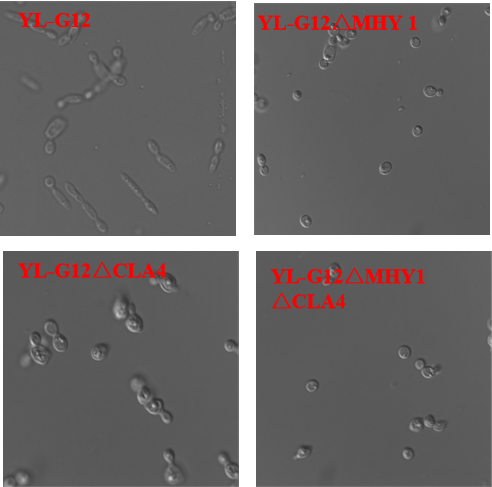

Supplement: S13 Fig — Microscopic images of strains YL-G12, YL-G12△MHY1, YL-G12△CLA4, and YL-G12△MHY1△CLA4. (TIF) [file pbio.3002131.s013.tif]

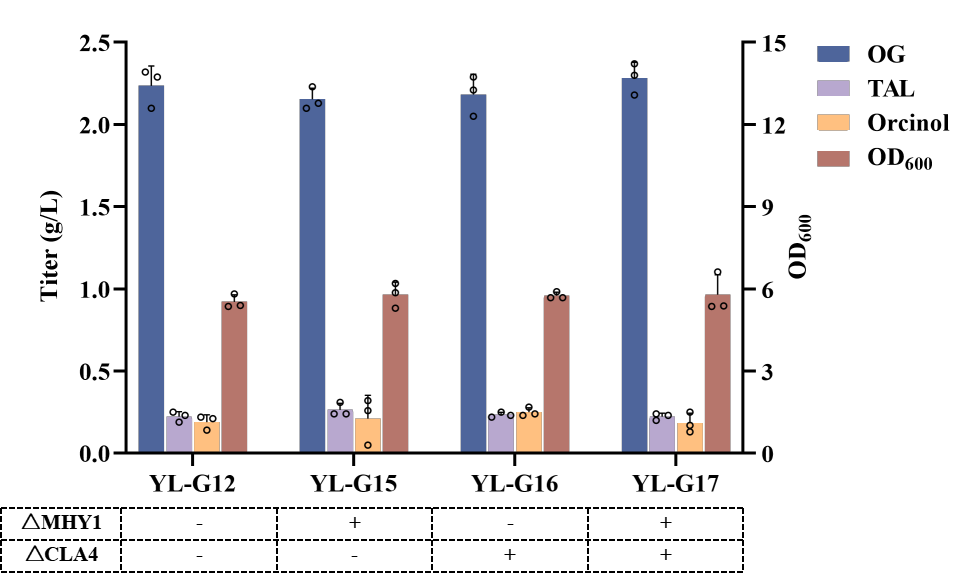

Supplement: S14 Fig — (TIF) [file pbio.3002131.s014.tif]

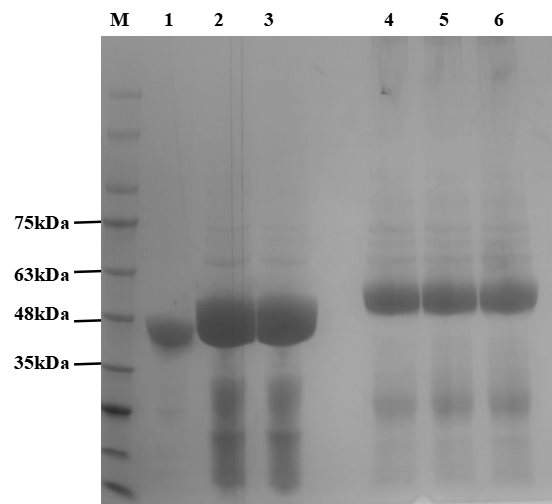

Supplement: S15 Fig — M: Marker, 1: CorcORS1, 2: RuauORS, 3: RdelORS1, 4: CorcUGT31, 5: CorcUGT32, 6: CorcUGT71. (TIF) [file pbio.3002131.s015.tif]

**Fig 1a**

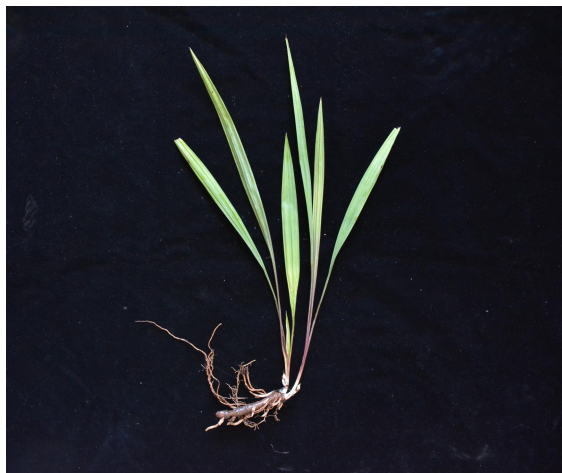

**Fig 2b**

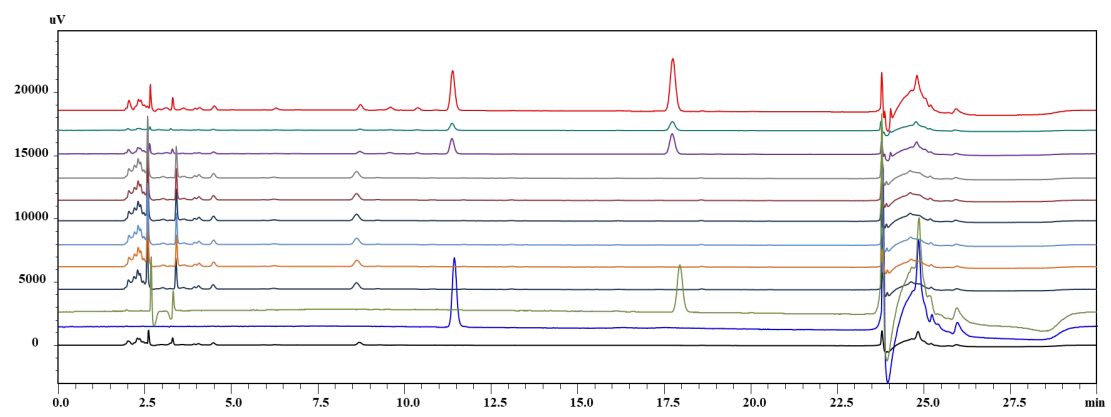

**Fig 3c**

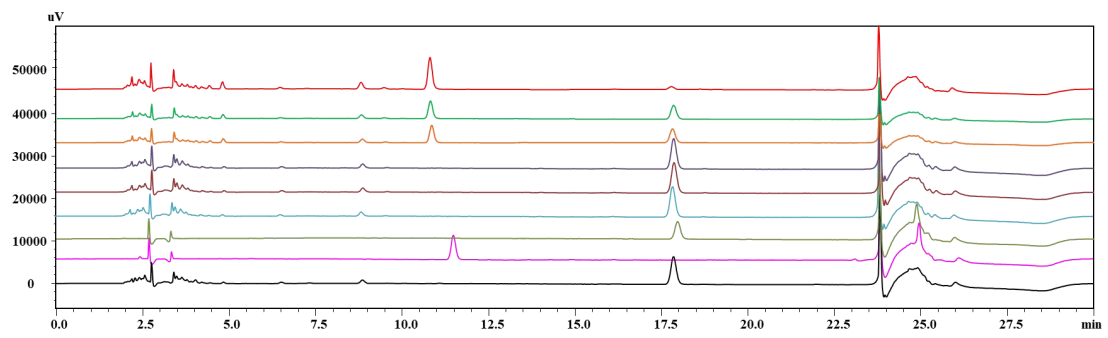

**Fig 3d**

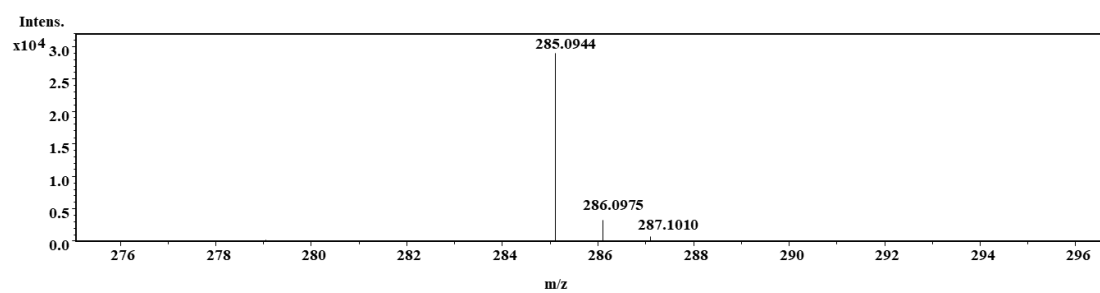

## S6 Fig

a

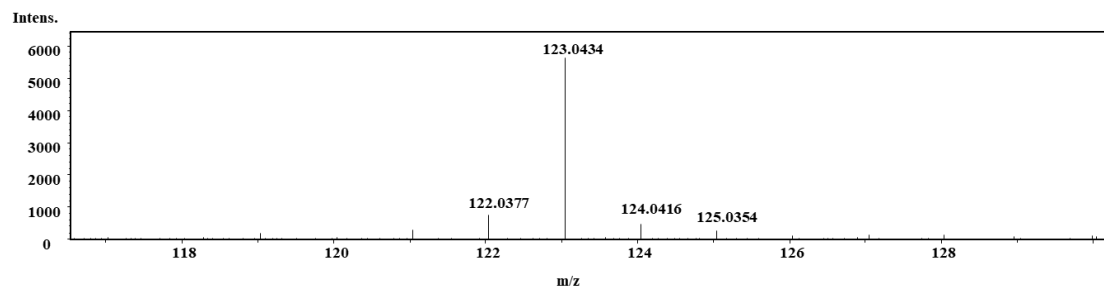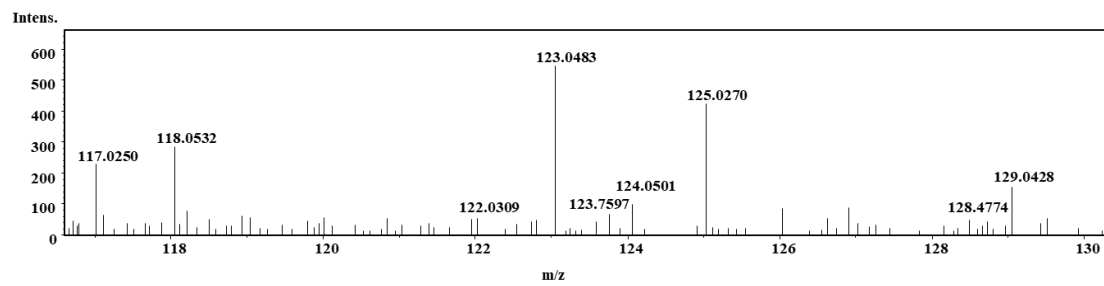

b

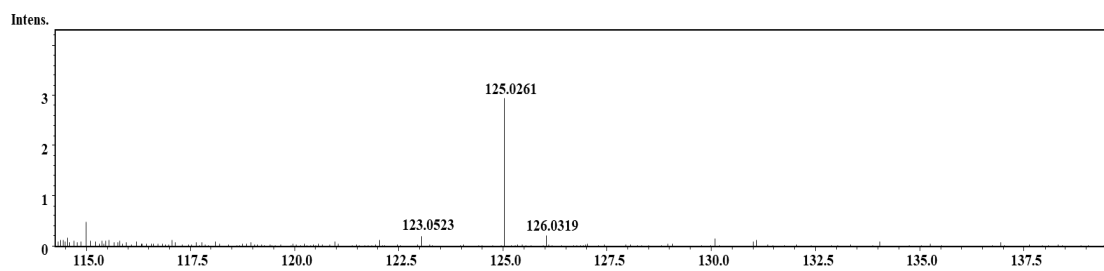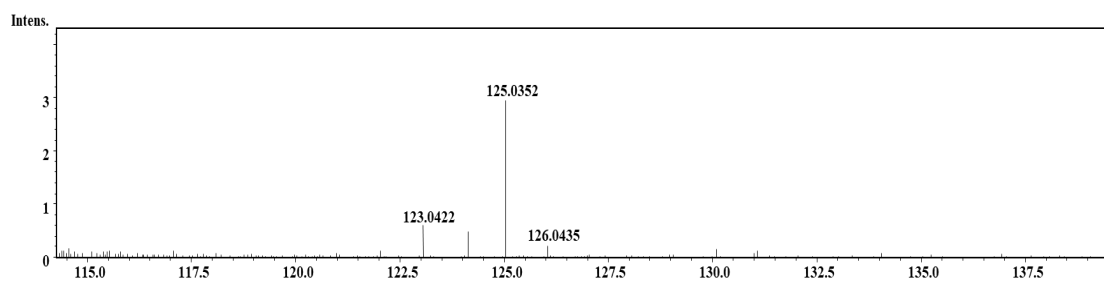

**S10 Fig**

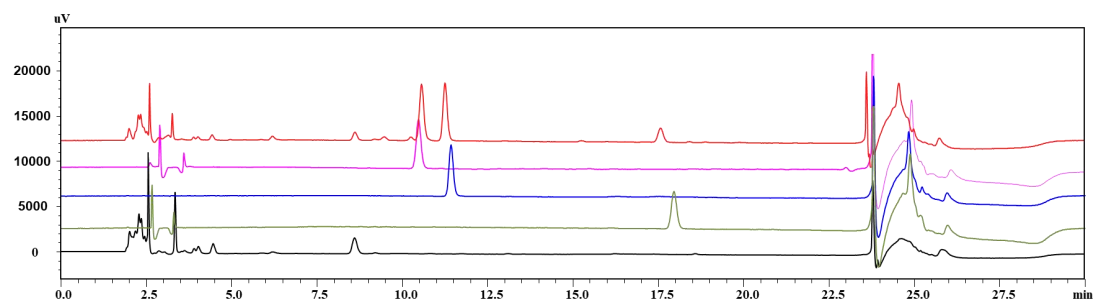

**S15 Fig**

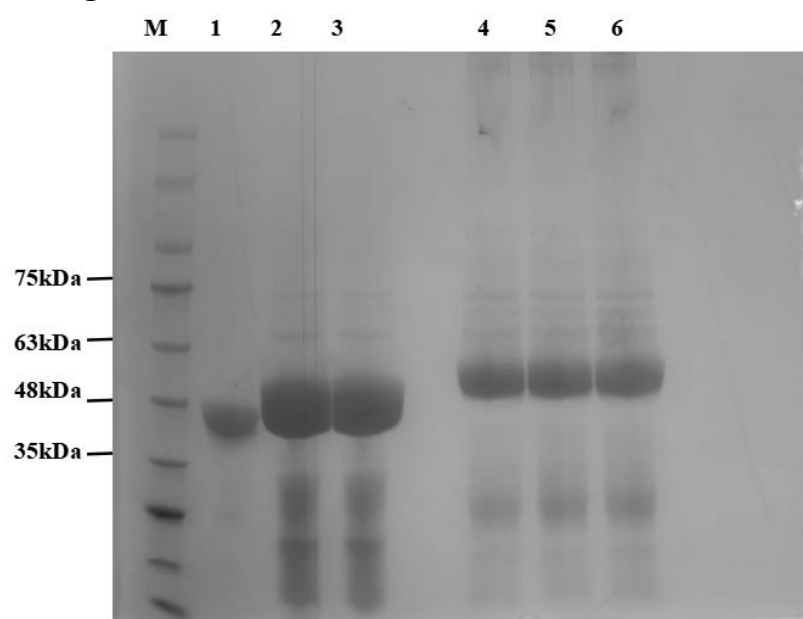

Supplement: S1 Raw Images — (PDF) [file pbio.3002131.s029.pdf]
