# Supplementary material for: Production of the antidepressant orcinol glucoside in Yarrowia lipolytica with yields over 6,400-fold higher than plant extraction
Source: PLoS Biol. 2023 Jun 6;21(6):e3002131. doi: 10.1371/journal.pbio.3002131 (PMC10243626; doi:10.1371/journal.pbio.3002131)
Supplement: S1 Text — (DOCX) [file pbio.3002131.s022.docx]

**S1 Text. Transcriptome data analysis of *C. orchioides.***

In total, 68,963 unigene pieces, the maximum length of 16,484 bp, the minimum length of 201 bp, the average length of 827.64 bp, GC content accounted for 43.84% and the N50 length was 1,489 bp (S1 Fig).

Gene Ontology annotation result shows, *C. orchioides* annotation to 50 GO-Term, include biological process cellular component and molecular function. Among them, 3,868 unigenes were annotated in biological process, 1,989 unigenes in cellular component and 12,165 unigenes in molecular function (S2 Fig). In the most representative secondary classification of molecular function, protein binding has 2,624 unigenes. According to the above annotation results, the gene function distribution of *C. orchioides* can be understood more clearly as a whole.

KEGG was used to analyze the results of 25,677 unigenes annotations of *C. orchioides* unigenes was mapped to 50 KEGG pathways, which could be divided into 6 categories according to the types of KEGG metabolic pathways. Among them, *C. Orchioides* unigenes is annotated the most in the Brite hierarchy (6,698 unigenes), contains almost all life activities, and is the least annotated in the cell process (111 unigenes). Among the 50 pathways, membrane traffickin involves the most genes (708 unigenes), while photosynthesis proteins and glyoxylate and dicarboxylate metabolism in eukaryotes involve the least genes, only 63 unigenes (S3 Fig). These data provide basic information for mining candidate genes involved in OG biosynthesis in *C. orchioides*.
