# Supplementary material for: Production of the antidepressant orcinol glucoside in Yarrowia lipolytica with yields over 6,400-fold higher than plant extraction
Source: PLoS Biol. 2023 Jun 6;21(6):e3002131. doi: 10.1371/journal.pbio.3002131 (PMC10243626; doi:10.1371/journal.pbio.3002131)
Supplement: S2 Text — (DOCX) [file pbio.3002131.s023.docx]

**S2 Text. Proportion of** **OG, orcinol and TAL inside and outside of cells.**

In order to detect OG, orcinol and TAL in the supernatant, the supernatant was collected by centrifugation at 12,000 rpm,15 min. In order to detect the OG, orcinol and TAL in the cells, the yeast cells were washed twice with double distilled water, extracted with 1 mL of 100% methanol, centrifuged at 12,000 rpm, 15 min.
