# Supplementary material for: Production of the antidepressant orcinol glucoside in Yarrowia lipolytica with yields over 6,400-fold higher than plant extraction
Source: PLoS Biol. 2023 Jun 6;21(6):e3002131. doi: 10.1371/journal.pbio.3002131 (PMC10243626; doi:10.1371/journal.pbio.3002131)
Supplement: S3 Text — (DOCX) [file pbio.3002131.s024.docx]

**S3 Text. Percent yield calculation for the fed batch experiment.**

1 mol glucose produces 2 mol acetyl-CoA, 1 mol acetyl-CoA produces 1 mol malonyl-CoA.

1mol OG needs 1 mol acetyl-CoA and 3 mol malonyl-CoA, and 1 mol UDP-glucose.

In theory, 3 mol of glucose is required to synthesize 1mol OG.

Thus, the theoretical yield is (1*286/3*180)*100%=52.96%

In the actual fermentation process, the initial medium: 400 mL (40 g/L glucose), the feed medium added 264.97 mL (600 g/L glucose).

Glucose consumption is 40 g*0.4 L+600 g*0.26497 L=174.98 g

After fermentation, the volume of fermentation liquid is only 600 mL, the OG titer is 43.46 g/L, Thus, the yield of OG is 43.46 g/L*0.6 L=26.08 g

Thus, the actual yield is (21.73 g/174.98 g)*100%=14.9%

Thus, the percent yield is (actual yield/theoretical yield)*100%=(14.9%/52.96%) *100%=28.13%

On the other hand：

The initial medium: 400 mL (40 g/L glucose), the DCW reached 16 g/L and glucose remaining 4.67 g/L at 24 h.

Glucose consumption is 40 g*0.4 L-4.67 g*0.4 L=14.13 g, the DCW is 16 g/L*0.4 L=6.4 g

Thus, this means that DCW length 1 consumes 14.13/6.4=2.2 g of glucose

After fermentation, the volume of fermentation liquid is only 600 mL, the DCW reached 51.67 g/L at 108 h, the DCW is 51.67 g/L*0.6 L=31 g

Thus, the cell growth consumes of glucose about 31*2.2=68.2 g of glucose

Thus, taking the glucose consumed by cell growth into account, actual yield is 26.08 g/ (174.98 g - 68.2 g)*100%=24.42%

Thus, the percent yield is (actual yield/theoretical yield)*100%=(24.42%/52.96%) *100%=46.11%
