# Supplementary material for: Production of the antidepressant orcinol glucoside in Yarrowia lipolytica with yields over 6,400-fold higher than plant extraction
Source: PLoS Biol. 2023 Jun 6;21(6):e3002131. doi: 10.1371/journal.pbio.3002131 (PMC10243626; doi:10.1371/journal.pbio.3002131)
Supplement: S4 Text — (DOCX) [file pbio.3002131.s025.docx]

**S4 Text. “Green and sustainable” calculation.**

In our system, the fermentation cycle is 4.5 days and the productivity of OG is 9.66 g/L/d in 1.3 L bioreactor. Currently, the planting cycle of *C. orchioides* is 5 years and the yield is about 4,500 kg/ha (~900 kg dry weight). The content of OG in the dry weight 0.131 mg/g and the yield is 0.1179 kg/ha. So the yield of OG for our strain in 1 cubic meter fermentor will be equal with the yield of OG for plants in 368.62 ha. The five-year output of fermentor is approximately equal to (365*5)/4.5*43.46≈17,601 kg, equivalent to 149,287 ha. In addition, compared with agricultural planting, it is economically feasible, saving more than 90% of fresh water resources, and avoiding the negative impact of pesticides and fertilizers on the environment.
