# Supplementary material for: Production of the antidepressant orcinol glucoside in Yarrowia lipolytica with yields over 6,400-fold higher than plant extraction
Source: PLoS Biol. 2023 Jun 6;21(6):e3002131. doi: 10.1371/journal.pbio.3002131 (PMC10243626; doi:10.1371/journal.pbio.3002131)
Supplement: S5 Text — (DOCX) [file pbio.3002131.s026.docx]

**S5 Text. Composition of media.**

Mineral medium containing 5 g/L (NH_4_)_2_SO_4_, 3 g/L KH_2_PO_4_, 0.5 g/L MgSO_4_·7H_2_O, 20 g/L D-glucose, 2 mL/L trace metal solution, 2 mL/L vitamin solution. The trace metals solution contained: 4.5 g/L CaCl_2_·2H_2_O, 4.5 g/L ZnSO_4_·7H_2_O, 3 g/L FeSO_4_·7H_2_O, 1 g/L H_3_BO_3_, 1 g/L MnCl_2_·4H_2_O, 0.4 g/L Na_2_MoO_4_·2H_2_O, 0.3 g/L CoCl_2_·6H_2_O, 0.1 g/L CuSO_4_·5H_2_O, 0.1 g/L KI, 15 g/L EDTA. The vitamin solution contained: 50 mg/L biotin, 200 mg/L p-aminobenzoic acid, 1 g/L nicotinic acid, 1 g/L Ca-pantothenate, 1 g/L pyridoxine-HCl, 1 g/L thiamine-HCl, 25 g/L myo-inositol. The feed medium contained 600 g/L of D-glucose, 25 g/L (NH4)_2_SO_4_, 15 g/L KH_2_PO_4_, 2.5 g/L MgSO_4_·7H_2_O, 10 mL/L trace metal solution, 10 mL/L vitamin solution. It is worth mentioning that in flask fermentation the D-glucose was 20 g/L in MM medium and in fermenter the D-glucose was set as 40 g/L in MM medium.

YNB medium composed of 1.7 g/L yeast nitrogen base, 5 g/L ammonium sulfate, 20 g/L glucose.

YPD medium consisting of 10 g/L yeast extract, 20 g/L peptone, 20 g/L glucose.
