# Supplementary material for: Production of the antidepressant orcinol glucoside in Yarrowia lipolytica with yields over 6,400-fold higher than plant extraction
Source: PLoS Biol. 2023 Jun 6;21(6):e3002131. doi: 10.1371/journal.pbio.3002131 (PMC10243626; doi:10.1371/journal.pbio.3002131)
Supplement: S6 Text — (DOCX) [file pbio.3002131.s027.docx]

**S6 Text. Methods for plasmids construction.**

**p1-CorcORS1-CorcUGT31 plasmid:** For the construction of p1-CorcORS1-CorcUGT31, the p1 plasmid was linearized by the amplification with primers p-T1-tPEX20-F/ p-T1-tlip2-F. The promoters TEFIN and GPD were obtained by the amplification from the genomic DNA of *Y. lipolytica* W29 with primers: p-T1-pTEFIN-F/R, p-T1-pGPD-F/R, respectively. Then the overlap-extension PCR of the two promoter fragments was performed to generate bidirectional promoters. The CorcORS1 and CorcUGT31 genes were amplified from the synthesized templates with primers p1-CorcORS1-F/R, p1-CorcUGT31-F/R, respectively. The resulted linearized plasmid, bidirectional promoter fragment, CorcORS1 fragment and CorcUGT31 fragment were then assembled by Gibson assembly method, yielding p1-CorcORS1-CorcUGT31 were introduced into DMT. The difference between p1-p6 was that the homologous arms of the integration sites were different and their promoters and terminators were the same pair. The other pn-xx-xx plasmids were constructed following the same protocol. n=1-6, xx represent any genes. The relative primers were listed in (S1 Table 1).

**pNat-YLgRNA** **series**: For the construction of G1, two fragments pgRNA-UP and pgRNA-DN were obtained by the amplification from pgRNA-YL with primers pgRNA-ZHONG-5F/G1-3R and pgRNA-ZHONG-3R/G1-5F, respectively. The resulted fragments were then assembled to generate G1 by Gibson assembly method. All the other pNat-YLgRNA plasmids were constructed following the same protocol. The relative primers were listed in (S1 Table 1).
