# Supplementary material for: Production of the antidepressant orcinol glucoside in Yarrowia lipolytica with yields over 6,400-fold higher than plant extraction
Source: PLoS Biol. 2023 Jun 6;21(6):e3002131. doi: 10.1371/journal.pbio.3002131 (PMC10243626; doi:10.1371/journal.pbio.3002131)
Supplement: S7 Text — (DOCX) [file pbio.3002131.s028.docx]

**S7 Text. Flask fermentation.**

YPD solid medium with hygromycin B (400 mg/L) and nourseothricin (250 mg/L) were used to screen the transformed *Y. lipolytica* strains. After 2 days of cultivation, individual colonies of recombinant strains were picked from plates, inoculated into 3 mL YNB in 24-well plates with gas permeable sealing membrane (Sigma-Aldrich) and grown for 48 h. The cultures were then transferred to a 250 mL shake flask containing 30 mL YNB medium and cultivated for 4 days. The yeast solution was streaked onto antibiotic-free YPD plates for 1 day to remove the gRNA plasmid in preparation for the next round of integration. All flask fermentation results represented the average standard deviation of three independent.
